# Microclimatic conditions mediate the effect of deadwood and forest characteristics on a threatened beetle species, *Tragosoma depsarium*

**DOI:** 10.1007/s00442-022-05212-w

**Published:** 2022-07-11

**Authors:** Ly Lindman, Erik Öckinger, Thomas Ranius

**Affiliations:** Department of Ecology, Swedish University of Agricultural Sciences, Box 7044, 750 07 Uppsala, Sweden

**Keywords:** Data-logger, Habitat, Moisture, Saproxylic, Temperature

## Abstract

While climate change has increased the interest in the influence of microclimate on many organisms, species inhabiting deadwood have rarely been studied. Here, we explore how characteristics of forest stands and deadwood affect microclimate inside deadwood, and analyse how this affects wood-living organisms, exemplified by the red-listed beetle *Tragosoma depsarium*. Deadwood and forest variables explained much of the variation in temperature, but less of the variation in moisture within deadwood. Several variables known to influence habitat quality for deadwood-dependent species were found to correlate with microclimate. Standing deadwood and an open canopy generates warmer conditions in comparison to downed logs and a closed canopy, and shaded, downed and large-diameter wood have higher moisture and more stable daily temperatures than sun-exposed, standing, and small-diameter wood. *T*.* depsarium* occupancy and abundance increased with colder and more stable winter temperatures, and with higher spring temperatures. Consistently, the species occurred more frequently in deadwood items with characteristics associated with these conditions, i.e. downed large-diameter logs occurring in open conditions. Conclusively, microclimatic conditions were found to be important for a deadwood-dependent insect, and related to characteristics of both forest stands and deadwood items. Since microclimate is also affected by macroclimatic conditions, we expect species’ habitat requirements to vary locally and regionally, and to change due to climate warming. Although many saproxylic species preferring sun-exposed conditions would benefit from a warmer climate per se, changes in species interactions and land use may still result in negative net effects of climate warming.

**Supplementary Information:**

The online version contains supplementary material available at 10.1007/s00442-022-05212-w.

## Introduction

Microclimate can by definition vary widely within small geographical areas. This has consequences for species distributions at multiple spatial scales, including their response to climate change (Suggitt et al. 2011). Modern low-cost data-loggers have made microclimatic data easier and cheaper to collect in the field (Terando et al. 2017), thus enabling studies to demonstrate the existence of strong links between microclimate and local biodiversity (De Frenne et al. 2021) as well as how microclimate is affected by topography (Gillingham 2010; Seidelmann et al. 2016), vegetation (Ohler et al. 2020), and land-use (Gillingham 2010).

Individual insect species typically perform optimally (survival, growth, fecundity, and dispersal) within a well-defined temperature range (Chown and Nicolson 2004). For that reason, the spatial distribution of microclimatically suitable habitats affects patterns of occurrence (Sillett et al. 2000). Outside the tropics, there is usually a part of the year when the temperature is too low for insect activity (Wolda 1988). Microclimate conditions then affect development times of insect life-stages and consequently their population dynamics (Johnson et al. 2016).

Approximately, one-third of all forest insect species are saproxylic, i.e. dependent on dying or dead trees (Ulyshen and Šobotnik 2018). Many of these species are declining in Europe at an alarming rate (Wallenius et al. 2010) because intensive forest management results in a lower amount and variability of deadwood (Siitonen 2001; Stokland et al. 2012; Gossner et al. 2013). Saproxylic species are often specialised on deadwood with certain qualities; wood diameter, direction, canopy openness, tree species, whether standing or downed, and stage of decay are all important characteristics (Siitonen et al. 2000; Stokland et al. 2012; Ranius et al. 2015; Buse et al. 2015). Several of these characteristics are assumed to influence the microclimate inside deadwood and consequently often used as proxies for microclimatic conditions (wood diameter: Schauer et al. 2018; direction: Burns et al. 2014; Buse et al. 2015; Müller et al. 2015; canopy openness: Seibold et al. 2016; Thorn et al. 2018; Müller et al. 2020), and log/snag: Bouget et al. 2012; Johansson et al. 2017). Only for certain characteristics, such as canopy openness, direction (Vermunt et al. 2012; Romo et al. 2019), and the depth of wood (Walczyńska and Kapusta 2017; Romo et al. 2019), there are indeed evidence that they are related with microclimate inside deadwood.

Since a long time, the effect of temperature and moisture on saproxylic insects has been studied (Graham 1925). It has been suggested that many of these species favour high (Stokland et al. 2012; Seibold et al. 2016; Lettenmaier et al. 2022) and stable (Warren and Key 1991; Gimmel and Ferro 2018) temperatures. Only recently, studies have combined species data with measurements of microclimate inside deadwood and found that species richness is positively related to average temperature (Henneberg et al. 2021). The internal microclimatic conditions, where larval development takes place, differ from those outside wood because wood buffers changes in temperature and moisture (Walczyńska and Kapusta 2017). However, the microclimatic conditions for saproxylic species have commonly been measured either in the air (e.g. Sheehan et al. 2019), near the ground (e.g. Sheehan et al. 2019), on the surface of logs (e.g. Kautz et al. 2013; Müller et al. 2015, 2020; Lettenmaier et al. 2022), or in the soil (e.g. Johansson et al. 2017), but not inside deadwood (see, however, Henneberg et al. 2021). This can make a big difference; for instance, the temperature near the surface of logs may vary much more than the ambient temperature (Graham 1925), while the opposite has been found deeper in the wood (Walczyńska and Kapusta 2017; Romo et al. 2019). Here, we explore microclimate variables measured inside deadwood (where insects develop), its correlation with forest and deadwood variables, and how all these variables explain the occurrence and abundance of a saproxylic beetle species.

We use a red-listed longhorn beetle, *Tragosoma depsarium* (Linnaeus, 1767), as a study species, because field observations indicate that it favours sun-exposed conditions (Wikars 2004; Swedish Species Information Centre 2020) and thus likely a warmer microclimate, even though the microclimatic requirements of this species have never been studied. This is a specialised species that occurs in boreal and alpine regions and is thus a representative for many saproxylic insects favouring sun-exposed conditions in these regions. Previous studies on microclimate of saproxylic insects have been conducted mainly on beetle assemblages under warmer conditions (Sheehan et al. 2019; Müller et al. 2015, 2020; Henneberg et al. 2021; Lettenmaier et al. 2022).

We hypothesise that the microclimatic conditions (temperature and humidity) inside deadwood are linked to characteristics of both the deadwood items and the surrounding forest and that this, in turn, can explain the occurrence patterns of saproxylic insects. Furthermore, we hypothesise that the preferred conditions of saproxylic insects are largely microclimatic, i.e. that both species’ abundance and occurrence patterns as well as habitat characteristics that explain these patterns are strongly correlated with microclimatic variables. The aim was to answer the following questions:

1. How are microclimatic conditions in deadwood affected by deadwood and forest characteristics known to affect saproxylic insects?
2. Which microclimatic variables within deadwood explain the occurrence and abundance of *T.* *depsarium*?
3. Which deadwood properties and forest characteristics explain the occurrence and abundance of *T.* *depsarium* and are they associated with favourable microclimatic conditions within deadwood?

## Materials and methods

### Study species

*T. depsarium* is a 20–35 mm long saproxylic longhorn beetle that occurs in Eurasia and North America (Foit 2007). In Asia, the species is found only in Siberia (Anisimov and Bezborodov 2021), while in Europe, it has a fragmented distribution from Sweden in the north to Greece in the south and from Spain in the west (Anisimov and Bezborodov 2021) to Russia in the east (Mannerkoski et al. 2010). The species inhabits coniferous forests (Foit 2007). In southern and central Europe, it is found in mountain regions, and in eastern Europe and Fennoscandia, in boreal forests (Mannerkoski et al. 2010).

In Fennoscandia, the larvae develop mainly in the sapwood (Bílý and Mehl 1989) of large-diameter, bark-free, and sun-exposed logs of Scots pine (*Pinus sylvestris*), or rarely Norway spruce (*Picea abies*) located in a dry or semi-dry environment (Palm 1951; Wikars 2004). The development takes 4 years or more (Palm 1951). When an adult emerges, it creates an easily identifiable large (up to 12 mm wide) oval exit-hole with rough edges, which cannot be mistaken for any other species on the Scandinavian mainland (Ehnström and Axelsson 2002; Gärdenfors et al. 2002). The species is reported to develop in quite recently dead trees, but also in logs which may have been dead more than 100 years (Palm 1951). As the *T. depsarium* is dependent on deadwood in open habitats, it is threatened by industrial forestry and fire-fighting practices, which are causing the forests to become younger and denser, and with less deadwood (Swedish Species Information Centre 2020). The species is classified as Near Threatened according to European Red List of the IUCN (Nieto and Alexander 2010) and as Vulnerable according to the Swedish national Red-List (Swedish Species Information Centre 2020).

### Study area and design

We conducted the study in south-eastern Sweden around 20 km northwest of Uppsala (Online Resource 1). The study landscape is around 250 km^2^ in size and contains large areas dominated by old pine forests that are only partly affected by clear-felling forestry (Länsstyrelsen i Uppsala län 2017).

**Fig. 1:**
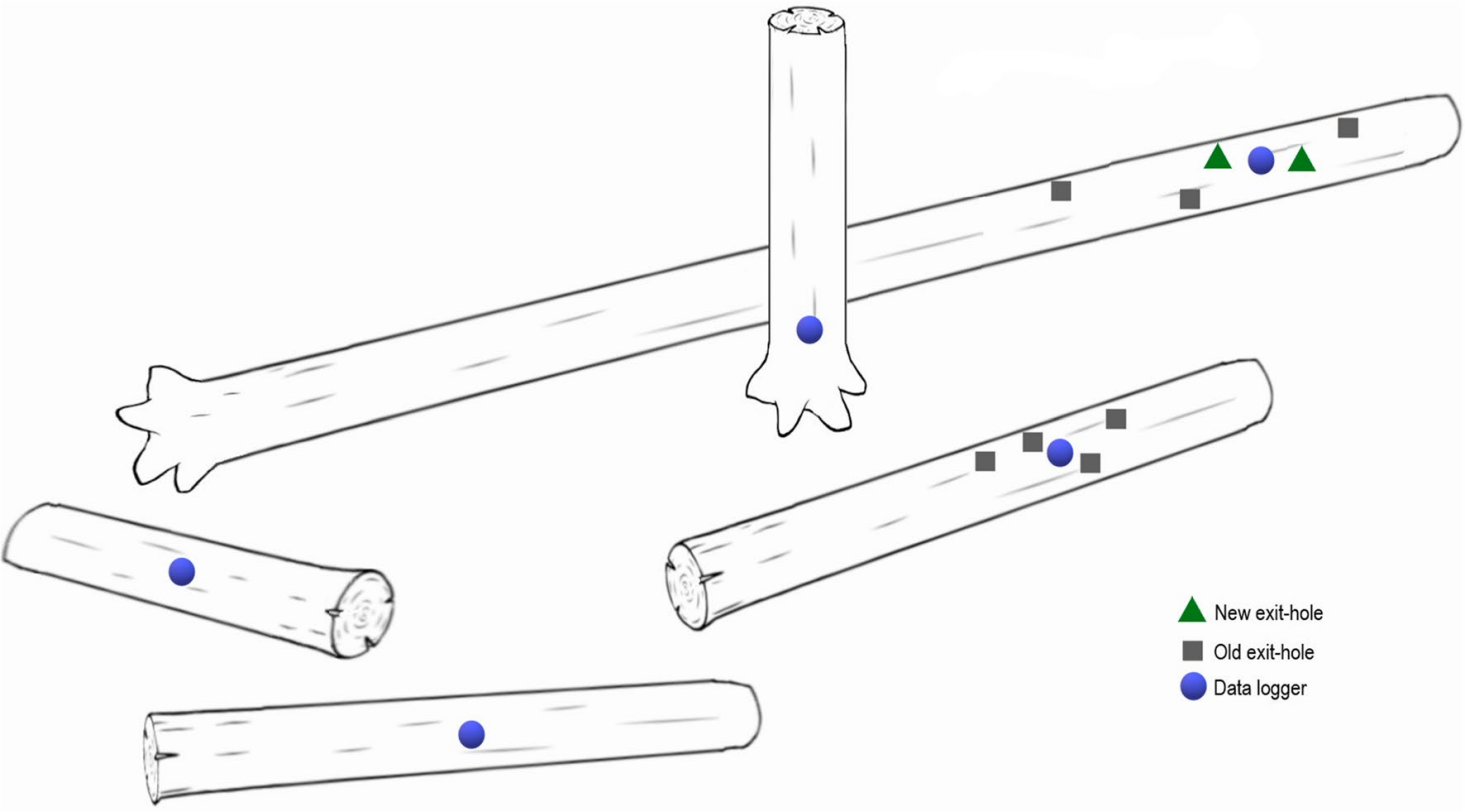
A schematic drawing of an identified study *site*, including four *logs* and one *snag*, with locations of new (green triangles) and old (grey squares) exit-holes and data-loggers (purple circles)

We studied the microclimate inside standing (*snag*) and downed (*log*) deadwood items of pine (*P. sylvestris*) with *T. depsarium* present or absent. The studied deadwood items were identified using data from a survey conducted in 2015 by Olof Hedgren (Länsstyrelsen i Uppsala län 2017), who reported the coordinates of 62 pine logs with recent exit-holes of the species in our study area. He surveyed thoroughly old (> 100 years) pine forest, clear-cuts, burned areas, and rocky outcrops, with the intention to record the species from as many sites as possible. We visited these logs in August and September 2019, searching for fresh exit-holes. New exit-holes are identifiable by their light creamy-white colour, like freshly cut wood; old exit-holes are dark and grey inside (see a picture in Online Resource 2). New exit-holes were from individuals that had emerged from the deadwood items in 2019 and 2020, while old exit-holes could be of individuals that had emerged many years earlier, but not later than 2018. When a deadwood item with a fresh exit-hole was found, a *site* was identified. It was defined as the deadwood item with a fresh exit-hole together with three or four closest deadwood items of pine (*snag* or *log*; Fig. 1), which never occurred farther away than 15 m. In a rare occasion, when two logs with a fresh exit-hole were found close together (not farther away than 15 m), both were included in the same *site*. Our intention with studying *sites* was to include items which potentially varied in their internal microclimatic conditions, but to minimise differences in colonisation rate caused by differences in spatial location. We identified 19 such *sites*. In addition, we included four *sites* where the species was absent as control *sites*, where four or five nearby deadwood pine items (not farther away than 15 m from each other) were defined as a *site*. We only included deadwood items with diameter ≥ 8 cm, because the species rarely uses smaller items (Wikars 2004). In total, 23 *sites* with 98 deadwood items were included in the study. For each deadwood item, we measured deadwood properties and forest characteristics at the location, and counted the number of new and old exit-holes of *T. depsarium*.

We used iButton Hygrochron DS 1923 (Maxim/Dallas Semiconductor Corp., USA) data-loggers to record temperature and relative humidity inside each deadwood item during the four seasons of a year (from October 2019 to September 2020). The loggers were placed inside deadwood items, on the most south-facing side (since all exit-holes were located on the most south-facing sides), either close to (1–3 cm) a new exit-hole or, if new exit-holes were absent, close to the old exit-holes or, if no exit-holes were present, in the middle of *logs* or 50 cm above the ground of *snags* (Fig. 1). The 50-cm level was chosen to reflect that wood of snags are, on average, situated higher from the ground than logs, but still we avoided a level so high that it would be lacking for some snags. In September 2019, we made holes 5 cm deep (to reach the sapwood, which for mature Scots pine occurs from the bark to 6–8 cm deep towards the centre; Bieker and Rust 2010) and 22 mm wide (according to the size of the loggers), placed loggers inside, and isolated them from the outside air with a wine cork and apple tree wax. *T. depsarium* often uses wood with existing galleries and exit-holes of this or other insects; 89% of new exit-holes were in deadwood items with old exit-holes of *T. depsarium*. Such galleries may affect the microclimate, but such an effect is impossible and not desirable to exclude from our measurements, since they are a part of the species’ environment. In addition, to measure ambient air temperature, we placed Lascar EL-USB-1 (Lascar Electronics, UK) temperature data-loggers in the middle of the *sites*, attaching them on the north side of trees at breast height (1.3 m from the ground).

We used hourly recorded microclimatic data inside deadwood from October (2019), January (2020), April (2020), and July (2020), to represent autumn, winter, spring, and summer, respectively. We calculated from the temperature data: (1) average temperature, (2) average daily temperature fluctuations (as a difference between daily minimum and maximum) in autumn, winter, spring and summer, and (3) temperature extremes (minimum in winter and maximum in summer); and from relative humidity data: (4) mean moisture in autumn, winter, spring and summer. Average temperature in summer was positively correlated to average temperature on spring and autumn, and negatively to winter temperature. Temperature fluctuations during different seasons were positively related to each other, and to summer maximum, and negatively to winter minimum. Moisture variables during different seasons were positively correlated to each other, but were not related to temperature variables (Online Resource 3). The 14 internal microclimate variables were chosen as temperature, moisture and their fluctuations at various parts of the year have been found to affect insects (Wolda 1988; Colinet et al. 2013). More specifically, for this species, average temperature could potentially be relevant as due to the preference of sun-exposure (Wikars 2004; Swedish Species Information Centre 2020). The effect of temperature fluctuations was tested since saproxylic insects are suggested to prefer stable microclimate (Warren and Key 1991; Gimmel and Ferro 2018). Temperature extremes were included because too high or too low temperature can be fatal for insects (Bale 1991). Moisture was tested since it is known to affect the activity of saproxylic insects (Graham 1924). The larval mortality may be affected by the conditions at any time of the year, while larval development time may be more affected by conditions at the warmer part of the year.

The winter temperature in 2020 was 3.3 °C, which was considerably higher than the average temperature during the last 30 years (− 2.6 ± 5.6 SD °C), while during other seasons the temperature was closer to the average (autumn 2019: 6.1 °C, 30-year average: 6.1 ± 4.4 SD °C; spring 2020: 5.7 °C, 30-year period: 5.3 ± 5.2 SD °C; summer 2020: 15.5 °C; 30-year period: 17.3 ± 4.7 SD °C; data from the closest weather station; SMHI 2021a).

### Deadwood and forest characteristics

For each deadwood item, we measured characteristics, which have been previously described as important for *T. depsarium* (Palm 1951; Wikars 2004) or which we assumed could influence microclimate (Online Resource 4). We divided them into two groups: (1) deadwood and (2) forest characteristics. All deadwood characteristics were measured either within a buffer zone of 2 m, either around the new exit-holes, or, if new exit-holes were absent, around the old exit holes or, if no exit-holes were present, in the middle of the deadwood items. The 2-m radius was chosen because often the old and new holes of *T. depsarium* are distributed over an area of that magnitude and the conditions are often homogenous within that distance. Forest characteristics were measured while standing above the downed *logs* and next to the *snags*.

#### Deadwood characteristics

We recorded whether the deadwood item was standing (*snag*) or downed on the forest floor (*log*). *Diameter* was measured with a calliper in the location of the logger. The *length* (or *height*) was measured with a measuring tape if the item was shorter than 2 m, while for longer/higher items it was estimated with 1 m accuracy. *Ground contact*, *bark cover* and *vegetation cover* of deadwood items were estimated as percentages of how much of the whole deadwood item was in contact with the ground, or covered by bark or vegetation, respectively.

For *softness*, we pressed a knife into the deadwood and measured the blade length that penetrated wood. This was done three times for each item and the average penetration was recorded.

#### Forest characteristics

To estimate *canopy openness*, photographs were taken with a fisheye lens ca 40 cm above the ground, placing the camera above *logs* or next to *snags*. For snags, photos were taken from two sides and combined after cutting off the halves including the snag itself. The photos were analysed with Gap Light Analyzer (Frazer et al. 1999). The variable expresses the percentage of the area not covered by canopy.

*Basal area*—the area (m^2^) of the cross-sections of the tree trunks at breast height (1.3 m) per hectare, commonly used as an indicator of stand density—was measured with a relascope while standing next to (or above if possible) each deadwood item. The value was estimated for each deadwood item because conditions may differ considerably within a *site*. The ratio between the chain length and the width of opening of the relascope was 1:35. To calculate *basal area*, the relascope counts were, therefore, multiplied by two (Järvis 2013).

The *vegetation types* were divided into three classes (Wikars 2004): dry (dominated by *Vaccinium vitis-idaea* L. and lichens on the ground); mesic (*V. myrtillus* L. and pleurocarpous mosses); and wet (Sphagnum-mosses in the bottom layer). However, no deadwood item was located in wet conditions.

We divided the *sites* into four *stand types*: (1) young clear-cut aged < 10 years, or a glade (diameter > 30 m), due to a thin soil layer—tree height usually ≤ 3 m; (2) old clear-cut aged around 11–24 years—tree height 3–15 m; (3) young forest aged around 25–75 years—tree height > 15 m, and average diameter < 20 cm; (4) mature forest aged > 75 years—tree height > 15 m, and average diameter > 20 cm.

Although topography has been found to affect microclimate (Gillingham 2010; Seidelmann et al. 2016), we did not include that variable since our study sites were located in a topographically homogenous landscape. Consistently, we only observed small differences in average air temperature between the warmest and coldest sites (between 0.8 °C in autumn and 2.7 °C in spring and summer).

### Statistical analysis

To analyse the effect of forest and deadwood characteristics (Online Resource 4) on internal microclimatic variables, we analysed each of the variables (the 14 variables of average temperature, temperature fluctuations and extremes, and mean moisture described above) as response variables in relation to (1) deadwood characteristics; (2) forest characteristics; and (3) deadwood and forest characteristics combined. We used Mixed ANOVA (linear mixed models) and included *site* as a random factor. Mean moisture values were transformed with Box–Cox transformation, analysed with linear models, and back-transformed when presenting the results.

To understand which variables explain the occurrence (presence/absence) and abundance of *T. depsarium*, we used four data sets: two of them reflected the current occurrence or abundance, and the remaining two the occurrence or abundance over a longer time span. The current occurrence was obtained by including all examined deadwood items, and considering the presence of new exit-holes. Long-term occurrence was the presence of all exit-holes (new and old) in all examined deadwood items. The current abundance was obtained by including only those deadwood items with new exit-holes present, and assessing the number of new exit-holes. Long-term abundance was obtained by assessing the number of exit-holes excluding the deadwood items with exit-holes absent.

We analysed the four response variables of current and long-term occurrence and abundance of *T. depsarium* in relation to deadwood and forest characteristics and internal microclimatic variables separately and together in order to find out which groups of variables best explain the patterns of *T. depsarium*. More precisely, we analysed these four response variables in relation to (1) deadwood characteristics; (2) forest characteristics; (3) deadwood and forest characteristics combined; (4) internal microclimatic variables (temperature and moisture); (5) deadwood and forest characteristics and internal microclimatic variables combined. Occurrence was analysed using generalised linear models with binomial distribution with presence (1) or absence (0) as the response variable. Abundance was analysed using generalised linear models with Poisson distribution. Variable *log/snag* was excluded from occurrence and abundance analyses because the species inhabited only *logs*.

We tested all possible models including maximum 4 variables at the time due to the small data set and high number of potential predictors (4 ≤ *k* ≤ 24, depending on the analysis), using second-order Akaike’s information criterion corrected for small sample size (AICc; R package *MuMIn* (Bartoń 2019)), as recommended when *N* (sample size) × *k* (number of predictors)^−1^ < 40 (Burnham and Anderson 2002). We considered all models with ΔAICc < 2 plausible (Burnham and Anderson 2002), and present them in supplementary material.

As we usually did not find a single best model, we performed model averaging to circumvent the problem of competing models. We extracted all possible models from a global model with a ∆AICc < 7 (Burnham et al. 2011) and performed multimodel inference (with the R package *MuMIn* (Bartoń 2019)) to calculate standardised averaged parameter estimates of all variables and estimated relative variable importance based on the sum of Akaike weights of all candidate models containing the variable. We considered relative variable importance > 0.5 important. Correlated variables (like several internal microclimatic variables; − 0.50 < *r* or *r* > 0.50) were never included in the same model.

As an absolute value for goodness-of-fit for the models of temperature variables, we reported *marginal* *R*^2^ [the proportion of variance explained by the fixed factor(s) alone] and *conditional* *R*^2^ (the proportion of variance explained by both the fixed and random factors, i.e. the variance explained by the whole model). For mean moisture, *adjusted* *R*^2^ values were presented for models of transformed data. Therefore, these were not included in comparisons with other microclimatic variables. To assess the predictive performance of our models of occurrence and abundance of *T. depsarium*, and to understand which variables are better at explaining the patterns of *T. depsarium*, we used *Nagelkerke’s pseudo-R-squared* (*R*^2^_*N*_) and coefficient of determination based on the *likelihood-ratio test* (*R*^2^_*LR*_) values, calculated separately for models with deadwood characteristics, forest characteristics, combination of deadwood and forest characteristics, microclimatic variables, and a combination of all the variables.

## Results

### Effect of deadwood and forest characteristics on the internal microclimate of deadwood

In comparison to the ambient air temperature, it was slightly warmer inside deadwood in autumn (5.7 ± 0.4 SD °C vs. 5.2 ± 3.7 °C) and summer (15.9 ± 1.2 °C vs. 15.7 ± 4.9 °C), while it was slightly colder in winter (1.7 ± 0.6 °C vs. 2.2 ± 2.8 °C) and spring (6.0 ± 1.2 °C vs. 6.1 ± 5.8 °C). The daily temperature fluctuations were always lower inside wood compared to the air (autumn: 3.2 ± 0.9 °C vs. 5.7 ± 3.0 °C; winter: 1.7 ± 0.7 °C vs. 4.0 ± 2.3 °C; spring: 8.4 ± 2.7 °C vs. 12.4 ± 6.5 °C; summer: 8.3 ± 3.0 °C vs. 10.7 ± 6.3 °C).

Deadwood and forest characteristics combined explained a considerable part of the internal average temperature in summer, autumn, and winter (0.33 ≤ *R*^2^_c_ ≤ 0.72), temperature fluctuations (0.26 ≤ *R*^2^_c_ ≤ 0.58), and temperature extremes (0.39 ≤ *R*^2^_c_ ≤ 0.47; Online Resources 5, 6). Deadwood characteristics explained internal average temperature in autumn and temperature fluctuations in autumn and winter better than forest characteristics. By contrast, forest characteristics were better at explaining average temperature in winter and summer, and temperature fluctuations in spring and summer (Online Resources 7, 8). Mean moisture was poorly (− 0.01 ≤ *R*^2^_adj_ ≤ 0.15) explained by deadwood and forest characteristics (Online Resource 6).

Four deadwood characteristics (especially *log* and *diameter*, but also *length* and *ground contact*) and three forest characteristics (especially *canopy openness*, but also *basal area*, and *vegetation type*) were relatively important in models predicting microclimate variables (Table 1).

**Table 1:** Model averaged parameter estimates (Est.), standard errors (SE), and relative variable importance (RVI) of deadwood and forest characteristics explaining differences in internal (1) average temperature, (2) daily temperature fluctuations, (3) temperature extremes, and (4) moisture in (a) autumn, (b) winter, and (c) spring, and (d) summer, based on models with ΔAICc < 7

Variables	Est	SE	RVI	Est	SE	RVI	Est	SE	RVI	Est	SE	RVI
1. Average temperature	(a) Autumn			(b) Winter			(c) Spring			(d) Summer		
Log/snag	− 0.311	0.129	**0.93**	− 0.389	0.086	**1.00**	− 0.041	0.143	0.20	− 0.055	0.148	0.23
Diameter							0.004	0.013	0.11	0.001	0.006	0.04
Ground contact	< 0.001	0.001	0.04									
Softness	0.001	0.005	0.02				< 0.001	0.004	0.01			
Canopy openness							0.005	0.010	0.18	0.043	0.007	**1.00**
Basal area				0.042	0.006	**1.00**						
Vegetation type	− 0.053	0.106	0.27	0.007	0.046	0.10	− 0.092	0.236	0.28	− 0.078	0.182	0.27
Stand type 2	− 0.005	0.041	0.02				− 0.037	0.166	0.13			
Stand type 3	− 0.010	0.080					− 0.108	0.404				
Stand type 4	− 0.004	0.037					− 0.085	0.267				
2. Daily temperature fluctuations	(a) Autumn			(b) Winter			(c) Spring			(d) Summer		
Log/snag	− 0.094	0.174	0.31	− 0.198	0.200	**0.59**	0.092	0.368	0.33	0.059	0.408	0.34
Diameter	− 0.050	0.012	**1.00**	− 0.016	0.016	**0.53**	− 0.004	0.017	0.06	− 0.066	0.060	**0.61**
Ground contact	< 0.001	0.001	0.03	− 0.010	0.002	**1.00**						
Softness	− 0.002	0.010	0.04	− 0.001	0.007	0.02	− 0.078	0.113	0.40	− 0.004	0.031	0.07
Canopy openness	0.028	0.005	**1.00**				0.079	0.027	**0.93**	0.105	0.017	**1.00**
Basal area				0.002	0.007	0.11						
Vegetation type	0.022	0.095	0.15	0.018	0.073	0.13	0.254	0.498	0.43	0.005	0.364	0.32
Stand type 2							− 0.092	0.388	0.07			
Stand type 3							− 0.097	0.617				
Stand type 4							− 0.214	0.809				
3. Temperature extremes				(b) Winter						(d) Summer		
Log/snag				− 0.092	0.127	0.42				− 0.012	0.266	0.27
Diameter				0.007	0.010	0.33				0.000	0.004	0.01
Softness										− 0.001	0.017	0.04
Canopy openness				− 0.007	0.008	0.45				0.100	0.014	**1.00**
Basal area				0.016	0.015	**0.55**						
Vegetation type				− 0.001	0.031	0.08				− 0.096	0.312	0.32
4. Moisture	(a) Autumn			(b) Winter			(c) Spring			(d) Summer		
Log/snag	89.34	78.68	**1.00**	− 35.02	55.15	0.20	− 37.60	56.58	0.20	38.07	48.33	0.29
Diameter	53.74	56.38	0.43	31.87	34.85	0.45	34.42	35.56	**0.56**	16.67	21.50	0.27
Length	− 27.40	31.46	0.21	8.41	11.68	0.22	13.45	13.80	**0.58**	4.45	5.91	0.26
Ground contact	31.78	42.77	0.15	− 19.51	23.70	0.29	11.81	20.94	0.16	8.50	12.97	0.20
Bark cover	20.03	44.10	0.15	14.89	23.32	0.20	− 19.37	24.43	0.23	− 8.70	13.88	0.18
Vegetation cover	48.63	50.49	0.46	16.70	24.62	0.21	22.29	26.48	0.29	15.79	17.86	0.42
Softness	59.52	62.67	0.41	22.69	36.69	0.21	23.04	35.95	0.18	21.09	27.72	0.26
Canopy openness	− 41.65	47.73	0.20	− 8.77	24.14	0.14	− 14.65	23.10	0.10	− 12.59	16.80	0.20
Basal area	40.24	49.80	0.14	27.12	31.95	0.31	30.88	33.51	0.44	20.23	22.88	0.40
Vegetation type	50.20	69.28	0.15	54.67	61.57	0.39	41.98	54.86	0.22	50.80	53.66	**0.54**
Stand type 2	32.08	58.57	< 0.01	− 12.39	37.83	< 0.01	16.79	38.05	< 0.01	11.22	31.13	0.02
Stand type 3	35.71	66.21		22.77	47.11		27.65	49.99		22.84	44.75	
Stand type 4	− 42.69	60.52		19.61	39.27		22.60	41.28		18.82	35.89	

For *stand* and *vegetation types*, the first categories are taken as references. Variable importance > 0.5 is highlighted in bold font

In comparison to *logs*, the average temperature was higher in *snags* (*snags*: autumn 5.9 ± 0.5 SD °C, winter 2.0 ± 0.6 °C; *logs*: autumn 5.6 ± 0.4 °C, winter 1.5 ± 0.5 °C), the daily temperature fluctuations were wider in winter (*snags*: 2.0 ± 0.8 SD °C; *logs*: 1.6 ± 0.6 °C), and the moisture lower in autumn (*snags*: 97.2 ± 7.5 SD %; *logs*: 101.5 ± 8.3%). In deadwood items with larger *diameters*, daily temperature fluctuations were lower in autumn, winter and spring, and the moisture higher in spring. Longer deadwood items had higher humidity in spring. With more *ground contact*, the temperature fluctuations were lower in winter (Table 1).

**Fig. 2:**
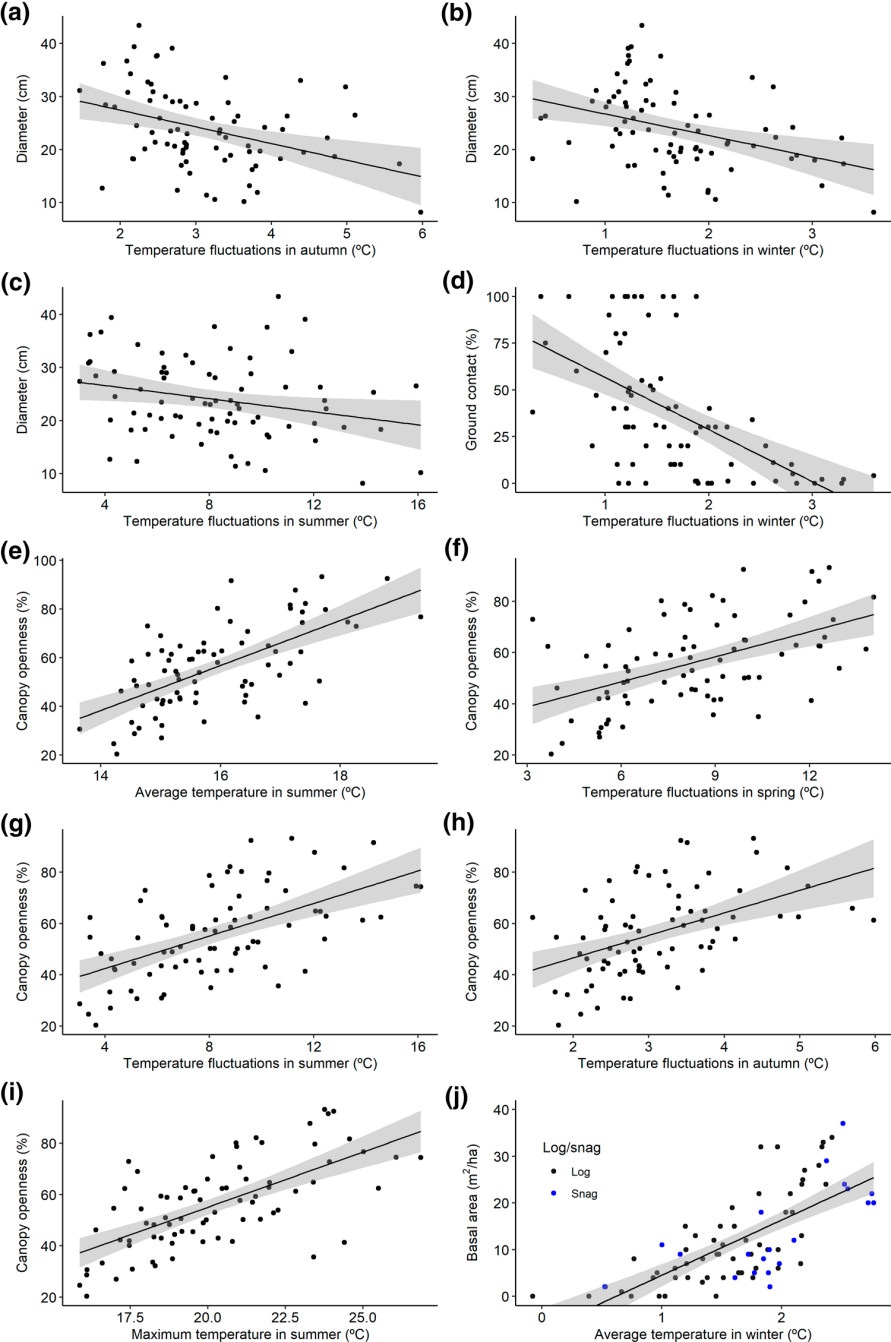
The relationships between diameter and temperature fluctuations in **a** autumn, **b** winter, and **c** summer; **d** ground contact and temperature fluctuations in winter; canopy openness and **e** average temperature in summer, temperature fluctuations in **f** spring, **g** summer, and **h** autumn, and **i** maximum temperature in summer; and **j** basal area and average temperature in winter in snags (blue symbols) and logs (black symbols). Trend line ± SE (as the grey area) are presented

*Canopy openness* affected thermal conditions during the warmer part of the year. Average temperature in summer (Fig. 2), daily temperature fluctuations in spring, summer and autumn, and summer maximum temperature increased with increasing *canopy openness* (Table 1). In turn, *basal area* had an effect on microclimate in winter. The average and minimum temperatures in winter increased with higher *basal area*. V*egetation type* affected moisture in summer, which was lower in dry *vegetation type* (in dry 85.4 ± 24.1 SD % versus in mesic 94.9 ± 15.8%).

### Occurrence and abundance of T. depsarium

We observed 472 exit-holes of *T. depsarium*. Among these, 100 exit-holes were new, distributed among 28 logs, while the 372 old exit-holes were distributed among 40 logs.

**Fig. 3:**
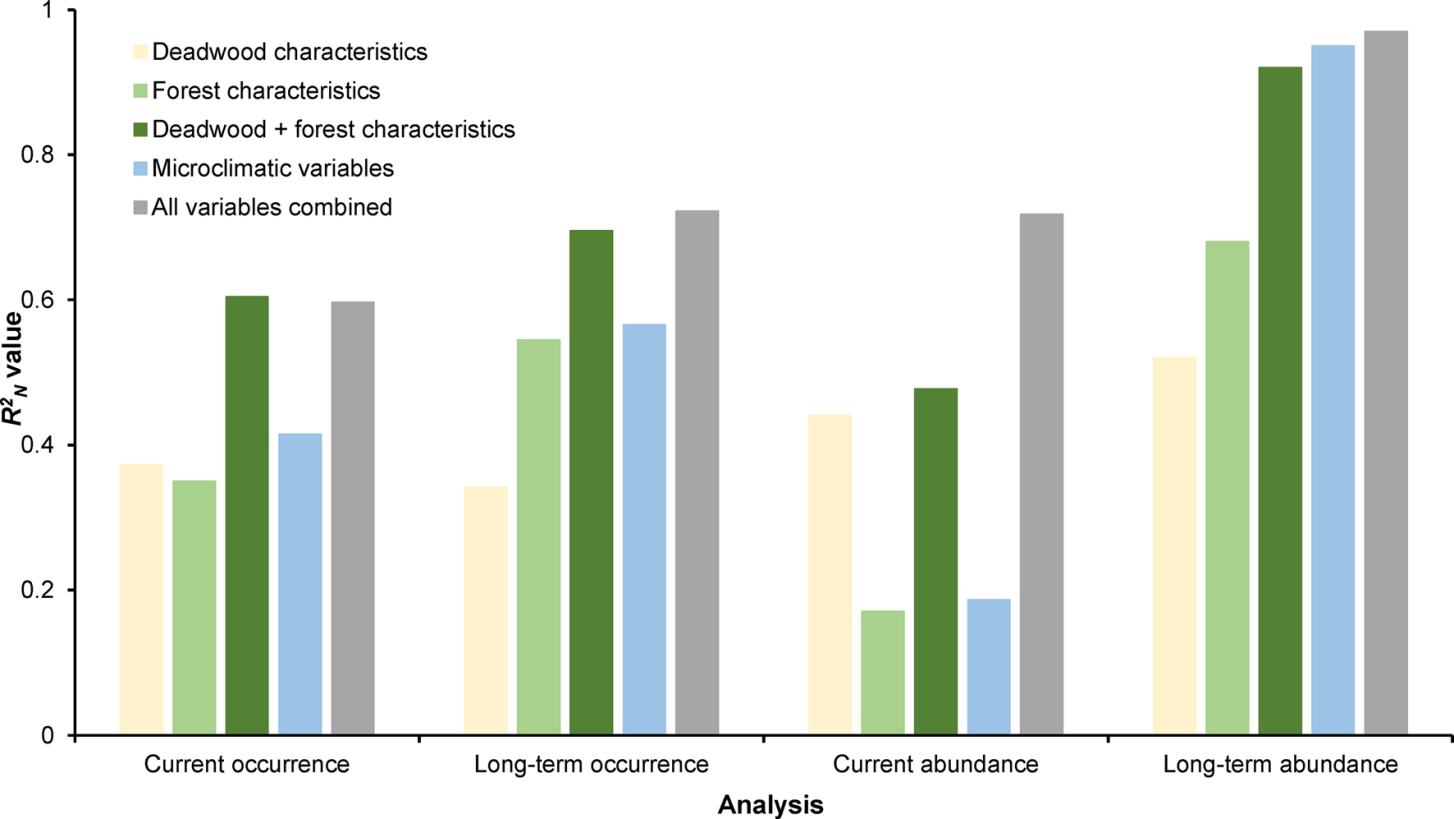
Predictive performance of models of current and long-term occurrence and abundance of *T. depsarium* in relation to deadwood characteristics, forest characteristics, the combination of deadwood and forest characteristics, microclimatic variables, and the combination of deadwood and forest characteristics and microclimatic variables

The combination of deadwood and forest characteristics and microclimatic variables was always better (0.54 ≤ *R*^*2*^_*N*_ ≤ 0.97) at explaining occurrence and abundance of *T. depsarium* (Fig. 3; Online Resources 9–11) than only deadwood characteristics (0.28 ≤ *R*^*2*^_*N*_ ≤ 0.52; Online Resource 12), only forest characteristics (0.17 ≤ *R*^*2*^_*N*_ ≤ 0.69; Online Resource 13), deadwood and forest characteristics combined (0.37 ≤ *R*^*2*^_*N*_ ≤ 0.92; Online Resource 14), or only microclimatic variables (0.09 ≤ *R*^*2*^_*N*_ ≤ 0.95; Online Resource 15). The combination of deadwood and forest characteristics explained current and long-term occurrence and current abundance better (0.37 ≤ *R*^*2*^_*N*_ ≤ 0.71) than microclimatic variables (0.09 ≤ *R*^*2*^_*N*_ ≤ 0.59), but slightly poorer long-term abundance of *T. depsarium* (*R*^*2*^_*N*_ = 0.92 vs. *R*^*2*^_*N*_ = 0.95). Current patterns (occurrence and abundance) were better explained by deadwood characteristics (0.37 ≤ *R*^*2*^_*N*_ ≤ 0.51) than forest characteristics (0.17 ≤ *R*^*2*^_*N*_ ≤ 0.35), while long-term patterns were better explained by forest characteristics (0.54 ≤ *R*^*2*^_*N*_ ≤ 0.69) than deadwood characteristics (0.28 ≤ *R*^*2*^_*N*_ ≤ 0.52).

*Diameter* was the most important variable explaining current and long-term occurrence and abundance (Table 2). *T. depsarium* occurred more likely and was more abundant in logs with bigger *diameter*.

**Table 2:** Model averaged parameter estimates (Est.), standard errors (SE), and relative variable importance (RVI) of (1) deadwood and forest characteristics, (2) microclimatic variables, and (3) combination of deadwood and forest characteristics, and microclimatic variables, explaining (a) current and (b) long-term occurrence, and (c) current and (d) long-term abundance, based on models with ΔAICc < 7

Variables		Est		SE	RVI	Est	SE	RVI	Est	SE	RVI	Est	SE	RVI
1. Deadwood and forest characteristics		(a) Current occurrence				(b) Long-term occurrence			(c) Current abundance			(d) Long-term abundance		
Diameter		0.279	0.085		**0.99**	0.254	0.084	**1.00**	0.048	0.039	**0.70**	0.045	0.014	**1.00**
Length		< 0.001	< 0.001		< 0.01				< 0.001	< 0.001	**0.56**			
Ground contact		< 0.001	0.004		0.17	0.001	0.005	0.19	− 0.001	0.002	0.19			
Bark cover		0.005	0.013		0.24	0.001	0.009	0.19	− 0.001	0.006	0.15			
Vegetation cover		0.002	0.009		0.18	− 0.001	0.010	0.18	< 0.001	0.003	0.14	− 0.006	0.005	**0.62**
Softness		− 0.079	0.134		0.39	0.003	0.062	0.18	0.072	0.065	**0.68**	0.120	0.022	**1.00**
Canopy openness		0.002	0.011		0.02				0.001	0.004	0.15	0.028	0.004	**1.00**
Basal area		− 0.173	0.061		**0.98**	− 0.228	0.064	**0.99**	0.002	0.012	0.14			
Vegetation type		− 0.217	0.564		0.26	− 0.597	0.894	0.45	0.115	0.223	0.32	− 0.149	0.207	0.38
Stand type 2						− 0.002	0.097	< 0.01	− 0.004	0.081	0.10			
Stand type 3						0.008	0.226		− 0.208	0.709				
Stand type 4						− 0.028	0.346		− 0.055	0.232				
2. Microclimatic variables		(a) Current occurrance				(b) Long-term occurrance			(c) Current abundance			(d) Long-term abundance		
Average temperature														
(A) Autumn		− 0.428		0.899	0.27	0.067	0.391	0.04	− 0.053	0.181	0.14	0.003	0.028	0.03
(B) Winter		− 0.085		0.411	0.05	− 2.616	1.967	**0.71**	− 0.046	0.175	0.16	− 0.664	0.186	**0.95**
(c) Spring		0.065		0.192	0.15	0.627	0.619	**0.62**	− 0.007	0.034	0.10	0.047	0.058	0.46
(d) Summer		0.164		0.326	0.26	0.240	0.471	0.26	− 0.029	0.076	0.17	0.013	0.037	0.14
Temperature fluctuations														
(a) Autumn		− 0.030		0.335	0.16	− 0.013	0.399	0.18	− 0.037	0.116	0.15	0.005	0.026	0.05
(b) Winter		− 2.318		1.013	**0.94**	− 0.687	1.181	0.29	− 0.088	0.206	0.26	− 0.016	0.078	0.07
(c) Spring		0.018		0.079	0.09	0.002	0.031	0.01	− 0.004	0.021	0.08	0.001	0.005	0.03
(d) Summer		0.010		0.052	0.08	− 0.029	0.097	0.16	− 0.003	0.018	0.08	0.001	0.007	0.05
Temperature extremes														
(a) Winter		− 0.075		0.349	0.13	− 0.551	1.255	0.20	− 0.016	0.182	0.12	− 0.032	0.145	0.05
(b) Summer		0.062		0.146	0.20	0.004	0.045	0.02	− 0.016	0.041	0.18	0.003	0.011	0.07
Moisture														
(a) Autumn		− 0.002		0.016	0.12	0.003	0.027	0.13	− 0.001	0.005	0.09	− 0.026	0.006	**1.00**
(b) Winter		0.006		0.013	0.30	− 0.007	0.015	0.30	0.003	0.007	0.28	0.015	0.004	**1.00**
(c) Spring		< 0.001		0.004	0.10	− 0.001	0.006	0.12	0.003	0.006	0.25			
(d) Summer		− 0.007		0.015	0.31	0.002	0.010	0.15	0.002	0.005	0.26			
3. All variables combined		(a) Current occurrence				(b) Long-term occurrence			(c) Current abundance			(d) Long-term abundance		
Diameter		0.274		0.093	**1.00**	0.281	0.118	**1.00**	0.040	0.067	0.33	0.109	0.028	**0.97**
Length									0.001	< 0.001	**0.72**			
Ground contact		< 0.001		0.003	0.06	0.001	0.005	0.07	< 0.001	0.001	0.03			
Bark cover		0.001		0.006	0.09	< 0.001	0.004	0.05	< 0.001	0.001	0.01			
Vegetation cover		0.001		0.007	0.09	0.005	0.020	0.10	− 0.003	0.008	0.12			
Softness		− 0.038		0.104	0.18	− 0.011	0.072	0.06	0.161	0.096	**0.81**	0.010	0.028	0.13
Canopy openness		0.002		0.011	0.04	0.002	0.013	0.02	< 0.001	0.001	0.02	0.015	0.010	**0.75**
Basal area		− 0.025		0.055	0.21	− 0.051	0.089	0.27	0.023	0.049	0.22	0.006	0.018	0.12
Vegetation type		− 0.156		0.544	0.13	− 0.893	1.582	0.32	0.033	0.149	0.07			
Stand type 2						0.003	0.896	0.21						
Stand type 3						− 2.965	1108							
Stand type 4						− 0.958	2.053							
Average temperature														
(a) Autumn		− 0.074		0.466	0.09	0.099	0.715	0.07	0.005	0.096	0.021	1.204	0.435	**0.92**
(b) Winter		− 0.757		1.095	0.39	− 0.911	1.563	0.30	− 0.265	0.516	0.27	− 0.065	0.229	0.08
(c) Spring		0.011		0.086	0.05	0.068	0.282	0.10	− 0.002	0.019	0.03			
(d) Summer		0.203		0.415	0.24	0.251	0.639	0.17	− 0.002	0.023	0.03			
Temperature fluctuations														
(a) Autumn		0.038		0.254	0.06	0.205	0.742	0.12	< 0.001	0.022	0.02			
(b) Winter		− 0.450		0.857	0.28	− 0.230	0.816	0.12	− 0.013	0.085	0.04			
(c) Spring		0.006		0.048	0.04	0.012	0.076	0.06	0.002	0.014	0.03			
(d) Summer		0.032		0.112	0.11	0.019	0.088	0.07	0.001	0.008	0.02			
Temperature extremes														
(a) Winter		− 0.089		0.436	0.06	− 0.738	1.761	0.17	− 0.436	0.845	0.27	− 1.777	0.577	**0.92**
(b) Summer		0.112		0.218	0.27	0.026	0.112	0.08	< 0.001	0.006	0.01	0.006	0.028	0.05
Moisture														
(a) Autumn		< 0.001		0.012	0.07	0.001	0.017	0.05	< 0.001	0.002	< 0.01	− 0.001	0.005	0.03
(B) Winter		< 0.001		0.005	0.07	− 0.013	0.025	0.28	0.001	0.005	0.11	0.001	0.003	0.03
(C) Spring		− 0.001		0.006	0.07	− 0.002	0.008	0.08	0.004	0.008	0.24			
(d) Summer		− 0.002		0.008	0.11	< 0.001	0.004	0.04	0.007	0.008	0.47			

For *stand* and *vegetation types*, the first categories are taken as references. Variable importance > 0.5 is highlighted in bold font

In comparison to unoccupied logs, occupied logs were situated in stands with smaller *basal area* (current: 6.4 ± 4.7 SD m^2^ ha^−1^ vs. 16.2 ± 10.5 m^2^ ha^−1^; long-term: 6.6 ± 4.8 m^2^ ha^−1^ vs. 19.4 ± 10.1 m^2^ ha^−1^; Table 2). In relation to microclimatic variables, daily temperature fluctuations in currently occupied logs were narrower in winter (1.2 ± 0.5 °C vs. 1.8 ± 0.6 °C). In long-term occupied logs, the average temperatures were slightly higher in spring (6.2 ± 1.4 °C vs. 5.5 ± 0.7 °C), but lower in winter (1.3 ± 0.5 SD °C vs. 1.9 ± 0.5 °C), in comparison to unoccupied logs.

Current and long-term *T. depsarium* abundance were larger in larger *diameter logs* with higher *softness* (Table 2). Current abundance was higher in *longer* logs, and long-term abundance was higher in more open *canopy* conditions with lower *vegetation cover*, higher average temperature in autumn and lower minimum temperature in winter. In relation to microclimatic variables, long-term abundance was higher in logs, where the winter temperature and autumn moisture were lower, and winter moisture higher.

## Discussion

During the last 2 decades, a large number of studies have revealed that different deadwood and forest characteristics (wood diameter, canopy openness, whether standing or downed, and decay stage) affect occurrence patterns of saproxylic insects (Stokland et al. 2012). At the same time, it is known that saproxylic insects are affected by the microclimatic conditions (Müller et al. 2020; Henneberg et al. 2021; Lettenmaier et al. 2022). We have shown a link between these factors: deadwood and forest characteristics important for saproxylic insects are driving microclimatic conditions inside deadwood. Furthermore, we have shown for the first time that microclimatic conditions inside deadwood explain a large part of the patterns of a saproxylic beetle species, and by including microclimatic variables in the models with deadwood and forest characteristics, the patterns are explained better than by any of the variable groups alone. This supports the view that insects are directly affected by the microclimatic conditions in deadwood.

### Importance of deadwood and forest characteristics for microclimate

The microclimate inside deadwood was affected by both deadwood and forest characteristics. The most important factors affecting microclimate in our study (Table 1) have all been found to be important for species inhabiting deadwood: habitat openness (*canopy openness* and *basal area*; Müller et al. 2015; Seibold et al. 2016), whether the deadwood item is standing or downed (Ranius et al. 2015), and the dimension of the item (*diameter*: Siitonen et al. 2000; Ranius et al. 2015; and *length*: Haeler et al. 2021).

During summer, and to some extent also spring and autumn, a higher degree of *canopy openness* implied higher average and maximum temperature as well as larger daily fluctuations. Similar temperature patterns were also found between sun-exposed and shaded logs (Lettenmaier et al. 2022). In contrast, during winter time, it was warmer where the *basal area* was higher. This is similar to observations of near-ground microclimate (Greiser et al. 2018), and can be explained by the fact that the canopy has a buffering effect on temperature (De Frenne et al. 2019) by reducing incoming solar radiation during the day and heat loss overnight (Geiger et al. 2012). The seasonal difference of importance of *canopy openness* and *basal area* may be due to lower effect of the sun radiation in winter time.

The average temperature was higher and fluctuated more in *snags* than in *logs* and snags were also drier. This can be because in downed *logs* the water content and temperature is buffered by the ground (Haughian and Frego 2017) and the vegetation often covering downed logs protects them from direct sunlight (Bässler et al. 2010). Note that for *snags*, we consistently measured the microclimate at a point 0.5 m from the ground, and microclimate may vary along the height of the snags.

Larger deadwood items—i.e. those with a higher *diameter,* and to some extent also greater *length*—had more stable temperatures and higher moisture. The higher moisture could be explained by a larger volume in relation to the surface area, and hence lower evaporation. The more stable temperatures could be due to that wood buffers temperature changes (Walczyńska and Kapusta 2017). Generally, higher moisture tends to decrease temperature fluctuations (Davis et al. 2019); however, in our data set, there was only a weak tendency to that (Online Resource 3). The more stable microclimatic conditions could be one reason why many wood-living species prefer deadwood of larger dimensions.

The difference in average temperature between the warmest and coldest deadwood items was between 2.2 °C (in autumn) and 5.7 °C (in summer). This is comparable with the predicted effect of climate warming in Uppsala (where this study was done), which is between 2.3 °C and 4.4 °C in the next 40 years according to an ensemble of nine climate scenarios based on RCP8.5, a scenario with emissions considerably higher than those laid down under the current Paris agreement (SMHI 2021a). It is also comparable with temperature differences between climatic regions. The observed difference between deadwood items corresponds to differences between sites in Sweden situated 400–1000 km from each other in a south-north direction (SMHI 2021b). From the southernmost and northernmost parts of Sweden, a distance of about 1750 km, the difference in annual average temperature is 9.6 °C. Thus, the variation between northern and southern Sweden is too large for *T. depsarium* to compensate for it by selecting different types of deadwood. Indeed, *T. depsarium* is absent from the coldest, northwestern part of Sweden (Swedish Species Information Centre 2020), probably because it is too cold.

### Occurrence and abundance of T. depsarium

*T. depsarium* occurred more often or at higher abundance in deadwood items with narrow temperature fluctuations and cooler temperatures during winter and with higher spring temperatures. Higher moisture in winter and lower in autumn was also favourable for the species. High temperatures and narrow temperature fluctuations are important for other species, e.g. a moth (Moore et al. 2021), and higher moisture for, e.g. dung beetles (Righi et al. 2018). The negative relationship with winter temperatures was unexpected, but an explanation could be that with decreasing winter temperatures, daily temperature fluctuations during this season decrease, and maximum in summer increased (Online resource 3). Greiser et al. (2018) observed similar seasonal patterns, due to that the more open areas that are colder in winter are warmer in summer.

We found that *T. depsarium* occurred more likely and abundantly in larger *diameter* wood and also was more abundant in *longer* logs. Furthermore, both current and long-term occurrence were more likely where the *basal area* was lower, and long-term abundance higher where *canopy openness* was higher and *vegetation cover* lower. The higher frequency of occurrence and abundance in larger logs could, except of more narrow temperature fluctuations, also be explained by more resources available. Higher *canopy openness*, and lower *basal area* and *vegetation cover* indicate open and sun-exposed, and therefore likely warmer conditions during the warmer part of the year. Thus, our results are agreeing with the habitat requirements of *T. depsarium* reported elsewhere (Palm 1951; Wikars 2004).

The forest and microclimatic variables were better at predicting long-term patterns than current patterns. One possible explanation is that the current patterns might be more dependent on the successional stage of the deadwood items and the occurrence of dispersal sources in recent years, while for long-term effects, forest characteristics and microclimatic conditions become relatively more important. In contrast, deadwood characteristics were better than microclimatic and forest stand variables at predicting current patterns. Thus, deadwood characteristics seem to change quicker, while forest characteristics and microclimatic conditions are more stable, and therefore more strongly associated with long-term patterns. The microclimatic variables were only measured during 1 year, but the strong relationship with long-term species patterns suggests that they are representatives for the microclimatic conditions over a much longer time. This may be because the relative microclimatic conditions in deadwood items may be similar between years, even though the absolute values change due to changing weather conditions.

## Implications for conservation

We have shown a strong correlation between internal microclimatic conditions in deadwood and the occurrence patterns of a specialised saproxylic beetle species. Furthermore, we found the microclimatic conditions to be explained by characteristics of the deadwood items and the forests, while others have shown that microclimate is related with local climatic conditions (De Frenne et al. 2021). Thus, we should expect saproxylic insect species to occur in different types of deadwood, and to have different levels of specialisation in different parts of their distribution areas, depending on local climatic conditions. Furthermore, we should expect that climate change will have a large impact on saproxylic organisms. They may not only change their distribution areas, but they may also remain within their current areas using deadwood with different characteristics, and thus with different microclimatic conditions.

Many saproxylic species, including our study species, prefer sun-exposed habitats (Graham 1924; Stokland et al. 2012; Seibold et al. 2016; Lettenmaier et al. 2022) and thus a warm microclimate. The status of such species would improve with climate warming in colder regions, as long as other factors are constant. However, changes in species interactions and land use may still result in negative net effects of climate warming.

Our study shows that the microclimate is affected by forest characteristics. Forest management for production tends to generate both more dense forests (Swedish Species Information Centre 2020) and, after clear-cutting, very sun-exposed conditions. It is well known that production forestry can have strong negative effects on saproxylic biodiversity, since it decreases the availability of deadwood (Siitonen 2000). Our study indicates that also the microclimatic conditions formed by forestry can be important for saproxylic insects.

## Supplementary Information

Below is the link to the electronic supplementary material.

Supplementary file1 (PDF 130 KB)

Supplementary file2 (PDF 48 KB)

Supplementary file3 (PDF 203 KB)

Supplementary file4 (PDF 27 KB)

Supplementary file5 (PDF 111 KB)

Supplementary file6 (PDF 425 KB)

Supplementary file7 (PDF 294 KB)

Supplementary file8 (PDF 432 KB)

Supplementary file9 (PDF 219 KB)

Supplementary file10 (PDF 230 KB)

Supplementary file11 (PDF 222 KB)

Supplementary file12 (PDF 225 KB)

Supplementary file13 (PDF 205 KB)

Supplementary file14 (PDF 251 KB)

Supplementary file15 (PDF 323 KB)

## Declarations

### Availability of data and materials

Available from the corresponding author on reasonable request.

### Code availability

Available from the corresponding author on reasonable request.
