# Supplementary material for: Microclimatic conditions mediate the effect of deadwood and forest characteristics on a threatened beetle species, Tragosoma depsarium
Source: Oecologia. 2022 Jul 11;199(3):737–52. doi: 10.1007/s00442-022-05212-w (PMC9309119; doi:10.1007/s00442-022-05212-w)
Supplement: Supplementary file 1 — Supplementary file1 (PDF 130 KB) [file 442_2022_5212_MOESM1_ESM.pdf]

Online Resource 1

Journal: Oecologia

Title: Microclimatic conditions mediate the effect of deadwood and forest characteristics on a threatened beetle species, *Tragosoma depsarium*

Authors: Ly Lindman, Erik Öckinger, Thomas Ranius

Corresponding author: L. Lindman, e-mail: Ly.Lindman@slu.se

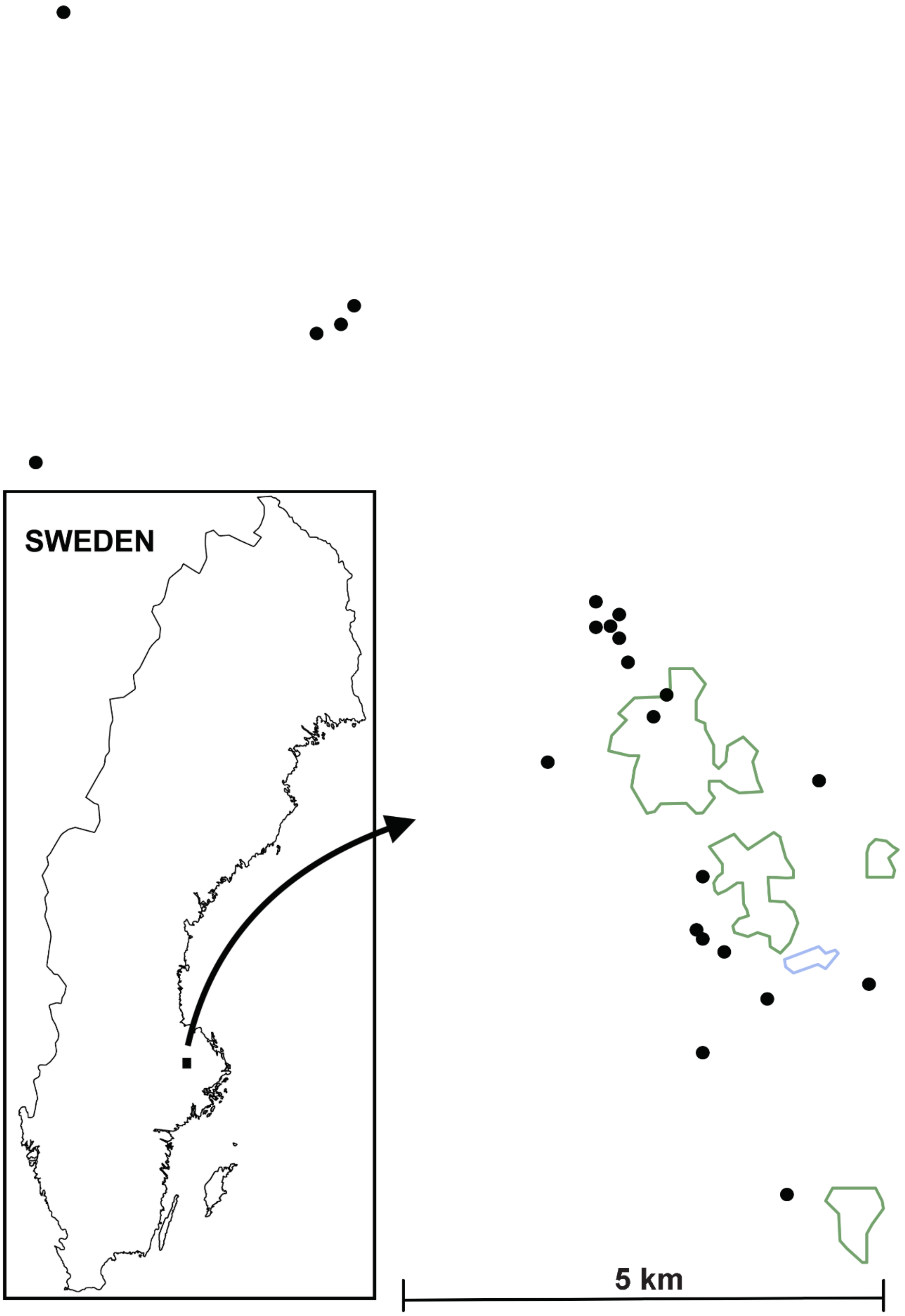

Online Resource 1 Location of study sites (black filled symbols) of *Tragosoma depsarium*, located close to four nature reserves (surrounded by green lines) and one conservation area (surrounded by blue line)
