# Supplementary material for: Microclimatic conditions mediate the effect of deadwood and forest characteristics on a threatened beetle species, Tragosoma depsarium
Source: Oecologia. 2022 Jul 11;199(3):737–52. doi: 10.1007/s00442-022-05212-w (PMC9309119; doi:10.1007/s00442-022-05212-w)
Supplement: Supplementary file 3 — Supplementary file3 (PDF 203 KB) [file 442_2022_5212_MOESM3_ESM.pdf]

Online Resource 3

Journal: Oecologia

Title: Microclimatic conditions mediate the effect of deadwood and forest characteristics on a threatened beetle species, *Tragosoma depsarium*

Authors: Ly Lindman, Erik Öckinger, Thomas Ranius

Corresponding author: L. Lindman, e-mail: Ly.Lindman@slu.se

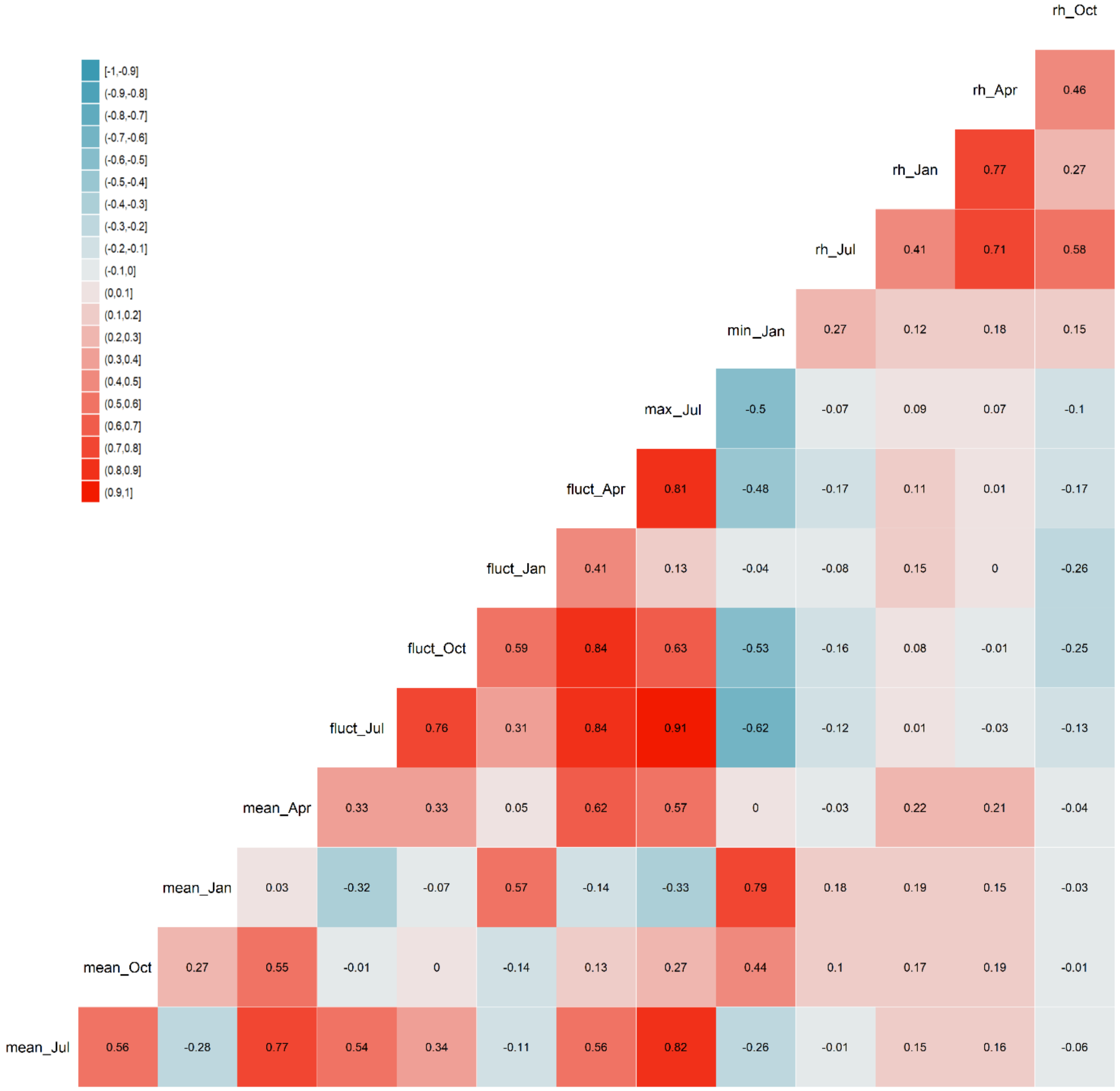

Online Resource 3 Correlation matrix of calculated internal microclimatic variables. Mean – average temperature, fluct – temperature fluctuations, max – maximum temperature, min – minimum temperature, rh – moisture, Jul – summer, Oct – autumn, Jan – winter, Apr – spring
