# Supplementary material for: Microclimatic conditions mediate the effect of deadwood and forest characteristics on a threatened beetle species, Tragosoma depsarium
Source: Oecologia. 2022 Jul 11;199(3):737–52. doi: 10.1007/s00442-022-05212-w (PMC9309119; doi:10.1007/s00442-022-05212-w)
Supplement: Supplementary file 4 — Supplementary file4 (PDF 27 KB) [file 442_2022_5212_MOESM4_ESM.pdf]

Online Resource 4

Journal: Oecologia

Title: Microclimatic conditions mediate the effect of deadwood and forest characteristics on a threatened beetle species, *Tragosoma depsarium*

Authors: Ly Lindman, Erik Öckinger, Thomas Ranius

Corresponding author: L. Lindman, e-mail: Ly.Lindman@slu.se

Online Resource 4 (a) Microclimatic variables, (b) deadwood, and (c) habitat characteristics used in the analyses. Minimum (Min), maximum (Max), mean (Mean) values and standard deviation (SD) of variables are presented. For details about measurements and calculations of the variables, see Methods

Variables	Description	Unit	Min	Max	Mean	SD
**a) Microclimatic variables**						
Average temperature	Calculated value, using data from temperature and humidity loggers					
a) in autumn		ºC	4.8	7.0	5.7	0.4
b) in winter		ºC	-0.1	2.8	1.7	0.6
c) in spring		ºC	3.0	8.6	6.0	1.2
d) in summer		ºC	13.7	19.4	15.9	1.2
Temperature fluctuations	Calculated value, using data from temperature and humidity loggers					
a) in autumn		ºC	1.5	6.7	3.2	0.9
b) in winter		ºC	0.3	3.6	1.7	0.7
c) in spring		ºC	3.2	15.0	8.4	2.7
d) in summer		ºC	3.0	16.1	8.3	3.0
Temperature extremes	Calculated value, using data from temperature and humidity loggers					
a) minimum in winter		ºC	-1.0	1.8	0.8	0.5
b) maximum in summer		ºC	15.9	26.9	20.3	2.6
Mean moisture	Calculated value, using data from temperature and humidity loggers					
a) in autumn		%	52.8	113	100	8.2
b) in winter		%	4.6	118	96.9	21
c) in spring		%	8.3	115	95.8	21
d) in summer		%	8.8	111	89.3	22
**b) Deadwood characteristics**						
Log/snag	Log when downed on the ground; snag when standing	1/0	0	1	0.7	0.4
Diameter	Diameter of deadwood items, measured with a calliper	cm	8.2	47.4	24.1	7.7
Length/height	Evaluated length of logs or height of snags	cm	30	2500	1031	709
Ground contact	Evaluated percentage of deadwood item touching the ground	%	0	100	37.1	34
Bark cover	Evaluated percentage of deadwood item covered by bark	%	0	90	12.8	23
Vegetation cover	Evaluated percentage of deadwood item covered by plants	%	0	90	14.7	20
Softness	Depth of knife penetration	cm	0.5	10	3.9	3.1
**c) Forest characteristics**						
Canopy openness	Based on fisheye photographs	%	20	93.2	55.6	16.6
Basal area	Calculated from relascope counts	$m^2\ ha^{-1}$	0	18.5	12.4	9.7
Vegetation type	Identified according to the plant species: (1) dry, (2) mesic, (3) wet	nr	1	2	1.5	0.5
Stand type	Identified according to the height and/or diameter of trees: (1) young and (2) old clear-cut (3) young and (4) mature forest	nr	1	4	2.3	1.2
