# Supplementary material for: Microclimatic conditions mediate the effect of deadwood and forest characteristics on a threatened beetle species, Tragosoma depsarium
Source: Oecologia. 2022 Jul 11;199(3):737–52. doi: 10.1007/s00442-022-05212-w (PMC9309119; doi:10.1007/s00442-022-05212-w)
Supplement: Supplementary file 5 — Supplementary file5 (PDF 111 KB) [file 442_2022_5212_MOESM5_ESM.pdf]

Online Resource 5

Journal: Oecologia

Title: Microclimatic conditions mediate the effect of deadwood and forest characteristics on a threatened beetle species, *Tragosoma depsarium*

Authors: Ly Lindman, Erik Öckinger, Thomas Ranius

Corresponding author: L. Lindman, e-mail: Ly.Lindman@slu.se

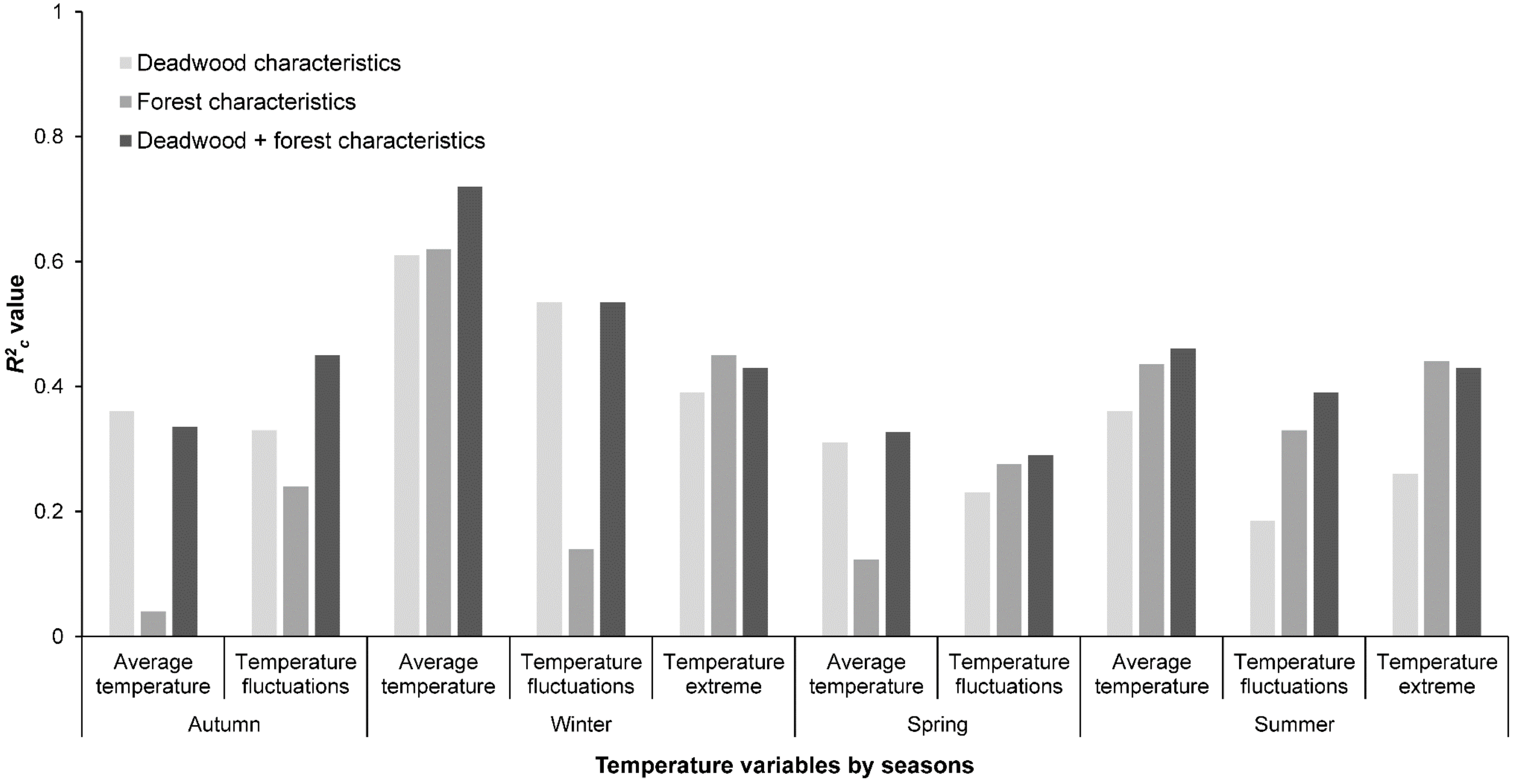

Online Resource 5 Predictive performance of models of average temperature and temperature fluctuations in autumn, winter, spring, and summer, and temperature extremes in winter and summer in relation to deadwood characteristics (light grey), forest characteristics (medium grey), and the combination of deadwood and forest characteristics (dark grey)
