# Supplementary material for: Microclimatic conditions mediate the effect of deadwood and forest characteristics on a threatened beetle species, Tragosoma depsarium
Source: Oecologia. 2022 Jul 11;199(3):737–52. doi: 10.1007/s00442-022-05212-w (PMC9309119; doi:10.1007/s00442-022-05212-w)
Supplement: Supplementary file 6 — Supplementary file6 (PDF 425 KB) [file 442_2022_5212_MOESM6_ESM.pdf]

Online Resource 6

Journal: Oecologia

Title: Microclimatic conditions mediate the effect of deadwood and forest characteristics on a threatened beetle species, *Tragosoma depsarium*

Authors: Ly Lindman, Erik Öckinger, Thomas Ranius

Corresponding author: L. Lindman, e-mail: Ly.Lindman@slu.se

Online Resource 6 Plausible candidate models (ΔAICc < 2) explaining (1) average temperature; (2) temperature fluctuations and (4) mean moisture in (a) autumn, (b) winter, (c) spring and (d) summer, and (3) temperature extremes in (a) winter and (b) summer, in relation to deadwood and forest characteristics. *Site* as a random factor is included in all models of temperature variables.). For *vegetation type*, the first category is taken as a reference. Sample size (N), intercept (Int.), number of parameters (k), model weight (w_i), *marginal* R^2 (R^2_m), *conditional* R^2 (R^2_c), and *adjusted* R^2 (R^2_{adj}) are presented

	N	Int.	log	dia-meter	length	ground contact	bark	veget. cover	soft-ness	canopy	basal area	veget. type	k	LogLik	ΔAICc	w_i	R^2_m	R^2_c	R^2_{adj}
**1. Average temperature**																			
a) autumn	76	5.91	-0.333										4	-43.2	0.00	0.71	0.11	0.33	
		6.00	-0.328									-0.200	5	-43.0	1.78	0.29	0.15	0.34	
b) winter	77	1.41	-0.389								0.042		5	-39.3	0.00	1.00	0.57	0.72	
c) spring	77	5.94											3	-117.4	0.00	0.52	0	0.31	
		6.11										-0.384	4	-116.9	1.25	0.28	0.03	0.34	
		4.55								0.025			4	-117.2	1.89	0.20	0.13	0.33	
d) summer	77	13.44								0.044			4	-106.6	0.00	0.72	0.4	0.45	
		13.73								0.041		-0.288	5	-106.4	1.91	0.28	0.41	0.47	
**2. Temperature fluctuations**																			
a) autumn	76	2.74		-0.049						0.027			5	-91.7	0.00	0.66	0.39	0.43	
		3.08	-0.300	-0.054						0.027			6	-91.2	1.36	0.34	0.41	0.47	
b) winter	77	3.02	-0.372	-0.031		-0.009							6	-67.7	0.00	0.62	0.42	0.58	
		2.09				-0.011							4	-70.6	0.98	0.38	0.30	0.49	
c) spring	77	3.75								0.081			4	-176.8	0.00	0.21	0.26	0.27	
		3.06								0.088		0.704	5	-175.7	0.09	0.21	0.27	0.29	
		4.25							-0.188	0.086			5	-175.9	0.45	0.17	0.31	0.31	
		3.54	0.284							0.081			5	-176.3	1.27	0.11	0.26	0.26	
		2.84	0.289							0.088		0.702	6	-175.2	1.42	0.11	0.27	0.28	
		3.82							-0.173	0.090		0.394	6	-175.3	1.59	0.10	0.31	0.31	
		4.02	0.325						-0.189	0.086			6	-175.3	1.74	0.09	0.31	0.31	
d) summer	77	4.94		-0.108						0.105			5	-181.8	0.00	0.40	0.39	0.41	
		2.44								0.104			4	-183.6	1.23	0.21	0.33	0.34	

Online Resource 6 Continued

	N	Int.	log	dia-meter	length	ground contact	bark	veget. cover	soft-ness	canopy	basal area	veget. type	k	LogLik	ΔAICc	w_i	R^2_m	R^2_c	R^2_{adj}
		4.96	-0.017	-0.108						0.105			6	-181.3	1.41	0.20	0.39	0.4	
		4.93		-0.108						0.106		-0.008	6	-181.4	1.48	0.19	0.39	0.41	
**3. Temperature extremes**																			
a) min in winter	77	1.92	-0.243							-0.016			5	-41.4	0.00	0.33	0.38	0.47	
		-0.05		0.021							0.031		5	-41.5	0.04	0.32	0.42	0.45	
		1.74								-0.016			4	-43.2	1.19	0.18	0.33	0.39	
		0.50									0.028		4	-43.2	1.28	0.17	0.31	0.41	
b) max in summer	77	14.57								0.101			4	-164.7	0.00	0.53	0.43	0.43	
		14.87								0.098		-0.302	5	-164.3	1.50	0.25	0.43	0.43	
		14.60	-0.042							0.101			5	-164.5	1.80	0.22	0.43	0.43	
**4. Mean moisture**																			
a) autumn	76	93.61	89.99	58.83				52.93					5	-2868.3	0.00	0.10			0.14
		96.85	88.66					52.70	65.04				5	-2868.4	0.13	0.09			0.14
		98.08	88.85					53.86					4	-2869.6	0.18	0.09			0.13
		97.58	88.07						66.66				4	-2869.7	0.51	0.08			0.12
		94.02	89.69	60.04									4	-2869.8	0.64	0.07			0.12
		93.65	89.29	58.25					64.78				5	-2868.7	0.84	0.07			0.13
		93.35	89.63	57.33				52.12	63.24				6	-2867.6	0.97	0.06			0.15
		93.98	91.52	59.48	-33.27			52.34					6	-2867.8	1.28	0.05			0.14
		94.42	91.62	60.61	-34.07								5	-2869.0	1.32	0.05			0.13
		99.15	88.67					52.50	66.02	-51.03			6	-2867.8	1.35	0.05			0.14
		99.89	88.10						67.50	-51.45			5	-2869.1	1.53	0.05			0.12
		95.88	90.03	58.98				52.90		-49.36			6	-2868.0	1.69	0.04			0.14
		95.43	88.92					52.76	66.80		53.33		6	-2868.1	1.84	0.04			0.14
		99.24	88.17										3	-2871.5	1.87	0.04			0.09
		98.55	90.05		-32.16			53.54					5	-2869.3	1.87	0.04			0.12

Online Resource 6 Continued

	N	Int.	log	dia-meter	length	ground contact	bark	veget. cover	soft-ness	canopy	basal area	veget. type	k	LogLik	ΔAICc	w_i	R^2_m	R^2_c	R^2_{adj}
		96.68	89.30	58.18					65.81	-51.36			6	-2868.1	1.93	0.04			0.13
		99.66	88.88					53.85		-48.84			5	-2869.3	1.93	0.04			0.12
b) winter	77	92.80		38.67							36.59		4	-1593.9	0.00	0.11			0.03
		100.53										67.40	3	-1595.0	0.02	0.10			0.02
		101.86											2	-1596.1	0.04	0.10			0.00
		95.11		37.24								68.05	4	-1594.0	0.23	0.09			0.03
		97.10		36.81									3	-1595.2	0.45	0.08			0.01
		100.08									34.79		3	-1595.2	0.52	0.08			0.01
		98.07		37.89		-26.48							4	-1594.4	1.05	0.06			0.02
		103.03				-25.39							3	-1595.5	1.12	0.06			0.01
		94.25		39.16		-24.98					35.55		5	-1593.4	1.37	0.05			0.03
		92.63		38.42							34.07	62.39	5	-1593.4	1.40	0.05			0.03
		99.74									31.36	63.53	4	-1594.7	1.73	0.05			0.01
		96.19		37.93		-24.67						65.54	5	-1593.6	1.73	0.05			0.02
		101.52				-23.21						65.43	4	-1594.8	1.80	0.04			0.01
		100.95			11.79								3	-1595.9	1.87	0.04			-0.01
		102.64	-53.86										3	-1596.0	1.99	0.04			-0.01
c) spring	77	89.19		40.11							37.79		4	-1595.4	0.00	0.14			0.06
		87.14		39.75	14.18						35.90		5	-1594.3	0.18	0.13			0.08
		90.90		38.26	15.04								4	-1595.6	0.37	0.12			0.06
		90.10		39.73			-27.29				38.49		5	-1594.8	1.07	0.08			0.07
		97.64			15.04								3	-1597.1	1.20	0.08			0.03
		86.41		38.87	14.51			28.47			36.04		6	-1593.7	1.26	0.07			0.08
		88.85		39.48				27.07			38.03		5	-1595.0	1.51	0.07			0.06
		90.37		37.25	15.31			28.25					5	-1595.0	1.51	0.07			0.06

Online Resource 6 Continued

	N	Int.	log	dia-meter	length	ground contact	bark	veget. cover	soft-ness	canopy	basal area	veget. type	k	LogLik	ΔAICc	w_i	R^2_m	R^2_c	R^2_{adj}
		96.18			15.38			29.75					4	-1596.2	1.55	0.06			0.04
		89.71		38.48	14.91							60.04	5	-1595.1	1.73	0.06			0.06
		86.01	58.23	40.86							38.17		5	-1595.1	1.76	0.06			0.06
		88.15		39.48	13.85		-26.01				36.79		6	-1594.0	1.77	0.06			0.07
d) summer	77	89.96						20.31				62.48	4	-1191.7	0.00	0.06			0.06
		92.02										60.81	3	-1193.1	0.46	0.05			0.04
		86.76			6.93			20.97				61.76	5	-1190.9	0.60	0.04			0.06
		88.83						20.14			26.40		4	-1192.1	0.68	0.04			0.05
		86.48	54.49					20.75				62.77	5	-1191.0	0.76	0.04			0.06
		87.95						20.56			23.39	57.25	5	-1191.0	0.84	0.04			0.06
		90.98									25.74		3	-1193.3	0.97	0.04			0.03
		84.57	56.12					20.67			26.85		5	-1191.1	1.10	0.03			0.06
		73.28	59.07	27.39							27.56		5	-1191.2	1.17	0.03			0.06
		71.44	60.17	26.31				19.88			27.96		6	-1190.0	1.18	0.03			0.07
		83.64	55.90					21.05			24.03	57.06	6	-1190.1	1.33	0.03			0.07
		82.94		25.34							26.80		4	-1192.4	1.39	0.03			0.04
		95.13						20.15		-18.32		59.43	5	-1191.3	1.43	0.03			0.05
		82.40			7.41			20.38	32.06			64.30	6	-1190.1	1.46	0.03			0.07
		86.75							32.85		27.76		4	-1192.5	1.49	0.03			0.04
		90.38									22.80	55.61	4	-1192.5	1.52	0.03			0.04
		85.27							34.16		25.37	57.86	5	-1191.3	1.53	0.03			0.05
		89.33							30.67			63.42	4	-1192.5	1.53	0.03			0.04
		89.78				15.51						63.30	4	-1192.5	1.59	0.03			0.04
		83.79						19.81	32.42		25.40	58.82	6	-1190.2	1.61	0.03			0.07
		89.41	52.01									60.95	4	-1192.5	1.62	0.03			0.04
		86.78		23.33								61.23	4	-1192.5	1.63	0.03			0.04
		88.03						19.78	28.29			64.20	5	-1191.4	1.64	0.02			0.05

Online Resource 6 Continued

N	Int.	log	dia-meter	length	ground contact	bark	veget. cover	soft-ness	canopy	basal area	veget. type	k	LogLik	ΔAICc	w_i	R^2_m	R^2_c	R^2_{adj}
	89.87			6.48							60.04	4	-1192.6	1.66	0.02			0.04
	97.27								-18.76		57.27	4	-1192.6	1.71	0.02			0.04
	101.26								-21.22			3	-1193.7	1.73	0.02			0.02
	86.19		21.34				19.80				62.63	5	-1191.4	1.73	0.02			0.05
	82.24		23.96				19.42			27.15		5	-1191.5	1.75	0.02			0.05
	85.51						19.43	30.96		27.90		5	-1191.5	1.85	0.02			0.05
	84.69			7.17				33.71			63.42	5	-1191.5	1.85	0.02			0.05
	87.78	53.69								26.10		4	-1192.7	1.87	0.02			0.03
	90.41						20.43				62.58	5	-1191.5	1.87	0.02			0.05
	89.17					-16.94	20.41			27.13		5	-1191.5	1.91	0.02			0.05
	79.31	63.04	23.68				20.14				57.33	6	-1190.4	1.98	0.02			0.06
