# Supplementary material for: Microclimatic conditions mediate the effect of deadwood and forest characteristics on a threatened beetle species, Tragosoma depsarium
Source: Oecologia. 2022 Jul 11;199(3):737–52. doi: 10.1007/s00442-022-05212-w (PMC9309119; doi:10.1007/s00442-022-05212-w)
Supplement: Supplementary file 7 — Supplementary file7 (PDF 294 KB) [file 442_2022_5212_MOESM7_ESM.pdf]

Online Resource 7

Journal: Oecologia

Title: Microclimatic conditions mediate the effect of deadwood and forest characteristics on a threatened beetle species, *Tragosoma depsarium*

Authors: Ly Lindman, Erik Öckinger, Thomas Ranius

Corresponding author: L. Lindman, e-mail: Ly.Lindman@slu.se

Online Resource 7 Plausible candidate models (ΔAICc < 2) explaining (1) average temperature; (2) temperature fluctuations and (4) mean moisture in (a) autumn, (b) winter, (c) spring and (d) summer, and (3) temperature extremes in (a) winter and (b) summer, in relation to deadwood characteristics. *Site* as a random factor is included in all models of temperature variables. Sample size (N), intercept (Int.), number of parameters (k), model weight (w_i), *marginal* R^2 (R^2_m), *conditional* R^2 (R^2_c), and *adjusted* R^2 (R^2_{adj}) are presented

	N	Int.	log	dia-meter	length	ground contact	bark	veget. cover	soft-ness	k	LogLik	ΔAICc	w_i	R^2_m	R^2_c	R^2_{adj}
**1. Average temperature**																
a) autumn	76	5.91	-0.333							4	-43.2	0.00	1.00	0.11	0.33	
b) winter	77	15.93								3	-118.1	0.00	0.63	0.00	0.34	
		16.19	-0.370							4	-117.6	1.07	0.37	0.02	0.38	
c) spring	77	5.94								3	-117.4	0.00	1.00	0.00	0.31	
d) summer	77	15.93								3	-118.1	0.00	0.63	0.00	0.34	
		16.19	-0.370							4	-117.6	1.07	0.37	0.02	0.38	
**2. Temperature fluctuations**																
a) autumn	76	4.25		-0.048						4	-98.4	0.00	0.62	0.15	0.33	
		4.66	-0.344	-0.054						5	-97.7	0.97	0.38	0.18	0.39	
b) winter	77	3.02	-0.372	-0.031		-0.009				6	-67.7	0.00	0.62	0.42	0.58	
		2.09				-0.011				4	-70.6	0.98	0.38	0.30	0.49	
c) spring	77	8.29								3	-183.0	0.00	0.66	0.00	0.23	
		8.28	0.023							4	-182.5	1.30	0.34	<0.01	0.23	
d) summer	77	8.26								3	-194.0	0.00	0.60	0.00	0.19	
		8.05	0.283							4	-193.3	0.85	0.40	<0.01	0.18	
**3. Temperature extremes**																
a) min in winter	77	0.84								3	-49.3	0.00	0.54	0.00	0.37	
		0.99	-0.220							4	-48.4	0.33	0.46	0.04	0.41	
b) max in summer	77	20.22								3	-180.3	0.00	0.64	0.00	0.26	
		20.43	-0.292							4	-179.7	1.16	0.36	<0.01	0.26	

Online Resource 7 Continued

	N	Int.	log	dia-meter	length	ground contact	bark	veget. cover	soft-ness	k	LogLik	ΔAICc	w_i	R^2_m	R^2_c	R^2_{adj}
**4. Mean moisture**																
a) autumn	76	93.61	89.99	58.83				52.93		5	-2868.3	0.00	0.13			0.14
		96.85	88.66					52.70	65.04	5	-2868.4	0.13	0.13			0.14
		98.08	88.85					53.86		4	-2869.6	0.18	0.12			0.13
		97.58	88.07						66.66	4	-2869.7	0.51	0.11			0.12
		94.02	89.69	60.04						4	-2869.8	0.64	0.10			0.12
		93.65	89.29	58.25					64.78	5	-2868.7	0.84	0.09			0.13
		93.35	89.63	57.33				52.12	63.24	6	-2867.6	0.97	0.08			0.15
		93.98	91.52	59.48	-33.27			52.34		6	-2867.8	1.28	0.07			0.14
		94.42	91.62	60.61	-34.07					5	-2869.0	1.32	0.07			0.13
		99.24	88.17							3	-2871.5	1.87	0.05			0.09
		98.55	90.05		-32.16			53.54		5	-2869.3	1.87	0.05			0.12
b) winter	77	101.86								2	-1596.1	0.00	0.26			0.00
		97.10		36.81						3	-1595.2	0.41	0.22			0.01
		98.07		37.89		-26.48				4	-1594.4	1.01	0.16			0.02
		103.03				-25.39				3	-1595.5	1.08	0.15			<0.01
		100.95			11.79					3	-1595.9	1.83	0.11			<0.02
		102.64	-53.86							3	-1596.0	1.95	0.10			-0.01
c) spring	77	90.90		38.26	15.04					4	-1595.6	0.00	0.28			0.06
		97.64			15.04					3	-1597.1	0.83	0.18			0.03
		90.37		37.25	15.31			28.25		5	-1595.0	1.14	0.16			0.06
		96.18			15.38			29.75		4	-1596.2	1.18	0.16			0.04
		94.64		38.24						3	-1597.5	1.64	0.12			0.02
		92.46	-56.90	37.50	15.51					5	-1595.4	1.95	0.10			0.05
d) summer	77	94.42								2	-1195.0	0.00	0.17			0.00
		93.02						19.31		3	-1194.1	0.36	0.14			0.01
		89.61			7.14			20.17		4	-1193.1	0.62	0.12			0.02

Online Resource 7 Continued

	N	Int.	log	diameter	length	ground contact	bark	veget. cover	softness	k	LogLik	ΔAICc	w_i	R^2_m	R^2_c	R^2_{adj}
		91.95			6.76					3	-1194.3	0.84	0.11			<0.01
		92.08	51.54							3	-1194.5	1.21	0.09			<0.01
		90.07	53.66					19.77		4	-1193.4	1.30	0.09			0.01
		90.11		22.67						3	-1194.6	1.35	0.09			<0.01
		94.78					-13.96			3	-1194.9	1.91	0.06			<0.01
		93.57							23.84	3	-1194.9	1.97	0.06			<0.01
		93.73				12.06				3	-1194.9	1.98	0.06			<0.01
