# Supplementary material for: Microclimatic conditions mediate the effect of deadwood and forest characteristics on a threatened beetle species, Tragosoma depsarium
Source: Oecologia. 2022 Jul 11;199(3):737–52. doi: 10.1007/s00442-022-05212-w (PMC9309119; doi:10.1007/s00442-022-05212-w)
Supplement: Supplementary file 9 — Supplementary file9 (PDF 219 KB) [file 442_2022_5212_MOESM9_ESM.pdf]

Online Resource 9

Journal: Oecologia

Title: Microclimatic conditions mediate the effect of deadwood and forest characteristics on a threatened beetle species, *Tragosoma depsarium*

Authors: Ly Lindman, Erik Öckinger, Thomas Ranius

Corresponding author: L. Lindman, e-mail: Ly.Lindman@slu.se

Online Resource 9 Plausible candidate models (ΔAICc < 2) explaining current occurrence in relation to combination of deadwood and forest characteristics and microclimatic variables (t ºC – average temperature, t ºC fluct. – daily temperature fluctuations, max t ºC – maximum temperature, RH % – relative humidity, aut. – autumn, sum. – summer). Sample size (N), intercept (Int.), number of parameters (k), model weight (w_i), a coefficient of determination based on the likelihood-ratio test (R^2_{LR}) and Nagelkerke's pseudo-R-squared (R^2_N) are presented

N	Int.	dia-meter	soft-ness	basal area	t ºC aut.	t ºC winter	t ºC sum.	t ºC fluct. winter	t ºC fluct. sum.	max t ºC sum.	RH % sum.	k	Log-Lik	ΔAICc	w_i	R^2_{LR}	R^2_N
55	-5.45	0.284			-1.977			-1.895		0.604		5	-20.0	0.00	0.15	0.47	0.63
	-16.79	0.296	-0.240			-1.934	0.837					5	-20.2	0.35	0.12	0.47	0.63
	-15.19	0.262				-1.661	0.693					4	-21.4	0.44	0.12	0.44	0.59
	-12.60	0.318				-1.734				0.355		4	-21.5	0.57	0.11	0.44	0.59
	-14.37	0.294	-0.242					-1.950		0.545		5	-20.4	0.81	0.10	0.46	0.62
	-13.91	0.271						-1.492		0.461		4	-21.7	0.96	0.09	0.43	0.59
	-12.45	0.343	-0.198			-2.073				0.387		5	-20.5	1.09	0.08	0.46	0.62
	-15.12	0.269				-1.532	0.782				-0.021	5	-20.9	1.74	0.06	0.45	0.61
	-7.46	0.276						-1.810	0.391			4	-22.2	1.90	0.06	0.43	0.57
	-2.73	0.238				-2.167						3	-23.3	1.91	0.06	0.40	0.54
	-12.60	0.301		-0.099						0.303		4	-22.2	1.96	0.05	0.42	0.57
