# Supplementary material for: Microclimatic conditions mediate the effect of deadwood and forest characteristics on a threatened beetle species, Tragosoma depsarium
Source: Oecologia. 2022 Jul 11;199(3):737–52. doi: 10.1007/s00442-022-05212-w (PMC9309119; doi:10.1007/s00442-022-05212-w)
Supplement: Supplementary file 10 — Supplementary file10 (PDF 230 KB) [file 442_2022_5212_MOESM10_ESM.pdf]

Online Resource 10

Journal: Oecologia

Title: Microclimatic conditions mediate the effect of deadwood and forest characteristics on a threatened beetle species, *Tragosoma depsarium*

Authors: Ly Lindman, Erik Öckinger, Thomas Ranius

Corresponding author: L. Lindman, e-mail: Ly.Lindman@slu.se

Online Resource 10 Plausible candidate models (ΔAICc < 2) explaining long-term occurrence in relation to combination of deadwood and forest characteristics and microclimatic variables (t ºC – average temperature, t ºC fluct. – daily temperature fluctuations, max t ºC – maximum temperature, min t ºC – minimum temperature, RH % – relative humidity, aut. – autumn, sum. – summer). For *vegetation* and *stand types*, the first categories are taken as references. Sample size (N), intercept (Int.), number of parameters (k), model weight (w_i), a coefficient of determination based on the likelihood-ratio test (R^2_{LR}) and Nagelkerke's pseudo-R-squared (R^2_N) are presented

**N**	**Int.**	**dia-meter**	**veget. cover**	**basal area**	**veget. type**	**stand 2**	**stand 3**	**stand 4**	**t ºC winter**	**t ºC spring**	**t ºC sum.**	**t ºC fluct. aut.**	**t ºC fluct. winter**	**min t ºC winter**	**max t ºC sum.**	**RH % winter**	**k**	**Log-Lik**	**ΔAICc**	w_i	R^2_{LR}	R^2_N
55	-21.39	0.275							-1.992		1.516					-0.052	5	-16.2	0.00	0.13	0.53	0.72
	-10.84	0.362			-2.768							3.164	-3.423				5	-16.2	0.03	0.13	0.53	0.72
	-25.72	0.345									1.673			-2.826		-0.060	5	-16.2	0.05	0.13	0.53	0.72
	-3.84	0.391			-3.665	1.294	-10.18	-3.881						-3.501			7	-13.7	0.30	0.12	0.57	0.77
	-3.78	0.374	0.077			-1.309	-1.545	-5.587						-4.014			7	-13.9	0.58	0.10	0.57	0.77
	-28.12	0.347			-2.018						1.775					-0.065	5	-16.7	1.04	0.08	0.52	0.71
	-1.40	0.223							-3.937	1.270						-0.043	5	-16.8	1.21	0.07	0.52	0.70
	-10.04	0.314		-0.162											0.438	-0.037	5	-16.8	1.34	0.07	0.52	0.70
	-3.04	0.231			-1.896				-3.256	0.733							5	-17.0	1.72	0.06	0.52	0.70
	0.79	0.240			-2.038				-3.088								4	-18.2	1.74	0.06	0.49	0.67
	-0.32	0.408				-1.102	-12.80	-5.198						-4.584		-0.035	7	-14.5	1.84	0.05	0.56	0.76
