# Supplementary material for: Microclimatic conditions mediate the effect of deadwood and forest characteristics on a threatened beetle species, Tragosoma depsarium
Source: Oecologia. 2022 Jul 11;199(3):737–52. doi: 10.1007/s00442-022-05212-w (PMC9309119; doi:10.1007/s00442-022-05212-w)
Supplement: Supplementary file 11 — Supplementary file11 (PDF 222 KB) [file 442_2022_5212_MOESM11_ESM.pdf]

Online Resource 11

Journal: Oecologia

Title: Microclimatic conditions mediate the effect of deadwood and forest characteristics on a threatened beetle species, *Tragosoma depsarium*

Authors: Ly Lindman, Erik Öckinger, Thomas Ranius

Corresponding author: L. Lindman, e-mail: Ly.Lindman@slu.se

Online Resource 11 Plausible candidate models (ΔAICc < 2) explaining (1) current and (2) long-term abundance in relation to combination of deadwood and forest characteristics and microclimatic variables (t ºC – average temperature, t ºC fluct. – daily temperature fluctuations, min t ºC – minimum temperature, RH % – relative humidity, aut. – autumn, sum. – summer). Sample size (N), intercept (Int.), number of parameters (k), model weight (w_i), a coefficient of determination based on the likelihood-ratio test (R^2_{LR}) and Nagelkerke's pseudo-R-squared (R^2_N) are presented

N	Int.	dia-meter	length	veget. cover	soft-ness	canopy	basal area	t ºC aut.	t ºC winter	min t ºC winter	RH % spring	RH % sum.	k	LogLik	ΔAICc	w_i	R^2_{LR}	R^2_N
**1. Current abundance**																		
23	-1.53		0.001		0.236				-0.756			0.016	5	-51.1	0.00	0.20	0.73	0.73
	-2.28		0.001		0.208							0.015	4	-53.0	0.49	0.16	0.68	0.69
	-1.59		0.001		0.246				-0.970		0.018		5	-51.4	0.55	0.15	0.72	0.73
	-2.60	0.166		-0.023			0.135			-2.204			5	-51.6	1.03	0.12	0.72	0.72
	-4.04	0.172					0.104			-2.352		0.014	5	-51.8	1.38	0.10	0.71	0.72
	-0.67	0.126		-0.029			0.126		-1.711				5	-51.8	1.40	0.10	0.71	0.72
	-1.98		0.001		0.222					-0.752		0.015	5	-51.8	1.47	0.09	0.71	0.72
	-4.52	0.169					0.115			-2.092	0.016		5	-52.0	1.83	0.08	0.71	0.71
**2. Long-term abundance**																		
33	-5.86	0.116				0.020		1.364		-1.975			5	-131.9	0.00	1.00	0.97	0.97
