# Supplementary material for: Microclimatic conditions mediate the effect of deadwood and forest characteristics on a threatened beetle species, Tragosoma depsarium
Source: Oecologia. 2022 Jul 11;199(3):737–52. doi: 10.1007/s00442-022-05212-w (PMC9309119; doi:10.1007/s00442-022-05212-w)
Supplement: Supplementary file 12 — Supplementary file12 (PDF 225 KB) [file 442_2022_5212_MOESM12_ESM.pdf]

Online Resource 12

Journal: Oecologia

Title: Microclimatic conditions mediate the effect of deadwood and forest characteristics on a threatened beetle species, *Tragosoma depsarium*

Authors: Ly Lindman, Erik Öckinger, Thomas Ranius

Corresponding author: L. Lindman, e-mail: Ly.Lindman@slu.se

Online Resource 12 Plausible candidate models (ΔAICc < 2) explaining (1) current and (2) long-term abundance, and (3) current and (4) long-term abundance in relation to deadwood characteristics. Sample size (N), intercept (Int.), number of parameters (k), model weight (w_i), a coefficient of determination based on the likelihood-ratio test (R^2_{LR}) and Nagelkerke's pseudo-R-squared (R^2_N) are presented

N	Int.	dia-meter	length	ground contact	bark	veget. cover	soft-ness	k	LogLik	ΔAICc	w_i	R^2_{LR}	R^2_N
**1. Current occurrence**													
71	-5.86	0.233						2	-36.7	0.00	0.44	0.27	0.37
	-5.63	0.227			-0.012			3	-36.4	1.61	0.20	0.28	0.38
	-5.94	0.231				0.011		3	-36.5	1.74	0.18	0.28	0.37
	-6.04	0.232		0.005				3	-36.5	1.80	0.18	0.28	0.37
**2. Long-term occurrence**													
71	-4.54	0.180					0.212	3	-38.3	0.00	0.20	0.25	0.34
	-3.47	0.175			-0.030			3	-38.4	0.33	0.17	0.25	0.34
	-3.93	0.174		0.012	-0.031			4	-37.3	0.35	0.16	0.27	0.37
	-4.09	0.175			-0.020		0.155	4	-37.5	0.72	0.14	0.27	0.36
	-4.79	0.180		0.008			0.187	4	-37.8	1.23	0.11	0.26	0.35
	-4.33	0.175		0.010	-0.024		0.119	5	-36.8	1.68	0.08	0.28	0.38
	-4.24	0.180		0.011				3	-39.2	1.97	0.07	0.23	0.31
	-3.81	0.181						2	-40.3	1.98	0.07	0.21	0.28
**3. Current abundance**													
29	-1.10	0.073					0.076	3	-75.5	0.00	0.27	0.43	0.43
	-1.26	0.059	<0.001				0.088	4	-74.1	0.00	0.27	0.48	0.48
	-1.02	0.082						2	-77.0	0.58	0.20	0.37	0.37
	-1.20	0.075	<0.001					3	-76.1	1.33	0.14	0.41	0.41
	-0.99	0.050	<0.001	-0.004			0.099	5	-73.4	1.50	0.13	0.51	0.51
**4. Long-term abundance**													
40	1.59	0.022				-0.008	0.073	4	-221.6	0.00	1.00	0.53	0.52
