# Supplementary material for: Microclimatic conditions mediate the effect of deadwood and forest characteristics on a threatened beetle species, Tragosoma depsarium
Source: Oecologia. 2022 Jul 11;199(3):737–52. doi: 10.1007/s00442-022-05212-w (PMC9309119; doi:10.1007/s00442-022-05212-w)
Supplement: Supplementary file 13 — Supplementary file13 (PDF 205 KB) [file 442_2022_5212_MOESM13_ESM.pdf]

Online Resource 13

Journal: Oecologia

Title: Microclimatic conditions mediate the effect of deadwood and forest characteristics on a threatened beetle species, *Tragosoma depsarium*

Authors: Ly Lindman, Erik Öckinger, Thomas Ranius

Corresponding author: L. Lindman, e-mail: Ly.Lindman@slu.se

Online Resource 13 Plausible candidate models (ΔAICc < 2) explaining (1) current and (2) long-term abundance, and (3) current and (4) long-term abundance in relation to forest characteristics. For *vegetation type*, the first category is taken as a reference. Sample size (N), intercept (Int.), number of parameters (k), model weight (w_i), a coefficient of determination based on the likelihood-ratio test (R^2_{LR}) and Nagelkerke's pseudo-R-squared (R^2_N) are presented

	N	Int.	canopy	basal area	veget. type	k	LogLik	ΔAICc	w_i	R^2_{LR}	R^2_N
**1. Current occurrence**											
	71	1.27		-0.159		2	-37.4	0.00	1.00	0.26	0.35
**2. Long-term occurrence**											
	71	2.84		-0.222		2	-30.4	0.00	0.63	0.40	0.54
		3.07		-0.216	-0.698	3	-29.8	1.03	0.37	0.41	0.55
**3. Current abundance**											
	29	1.06			0.463	2	-81.0	0.00	1.00	0.17	0.17
**4. Long-term abundance**											
	40	2.84		-0.061	-0.262	3	-213.3	0.00	0.72	0.69	0.69
		1.37	0.017		-0.271	3	-214.3	1.93	0.28	0.67	0.67
