# Supplementary material for: Microclimatic conditions mediate the effect of deadwood and forest characteristics on a threatened beetle species, Tragosoma depsarium
Source: Oecologia. 2022 Jul 11;199(3):737–52. doi: 10.1007/s00442-022-05212-w (PMC9309119; doi:10.1007/s00442-022-05212-w)
Supplement: Supplementary file 14 — Supplementary file14 (PDF 251 KB) [file 442_2022_5212_MOESM14_ESM.pdf]

Online Resource 14

Journal: Oecologia

Title: Microclimatic conditions mediate the effect of deadwood and forest characteristics on a threatened beetle species, *Tragosoma depsarium*

Authors: Ly Lindman, Erik Öckinger, Thomas Ranius

Corresponding author: L. Lindman, e-mail: Ly.Lindman@slu.se

Online Resource 14 Plausible candidate models (ΔAICc < 2) explaining (1) current and (2) long-term abundance, and (3) current and (4) long-term abundance in relation to forest characteristics. For *vegetation* and *stand types*, the first categories are taken as references. Sample size (N), intercept (Int.), number of parameters (k), model weight (w_i), a coefficient of determination based on the likelihood-ratio test (R^2_{LR}) and Nagelkerke's pseudo-R-squared (R^2_N) are presented

N	Int.	dia-meter	length	ground contact	bark	veget. cover	soft-ness	canopy	basal area	veget. type	stand 2	stand 3	stand 4	k	Log-Lik	ΔAICc	w_i	R^2_{LR}	R^2_N
**1. Current occurrence**																			
71	-4.93	0.266							-0.166					3	-27.5	0.00	0.28	0.44	0.59
	-4.16	0.277					-0.198		-0.186					4	-26.4	0.13	0.26	0.46	0.61
	-4.61	0.312					-0.235		-0.175	-0.990				5	-25.7	1.03	0.17	0.47	0.62
	-5.25	0.285			0.021				-0.191					4	-27.0	1.20	0.16	0.45	0.60
	-5.27	0.286							-0.157	-0.672				4	-27.1	1.53	0.13	0.44	0.60
**2. Long-term occurrence**																			
71	-2.59	0.267							-0.222	-1.305				4	-22.1	0.00	0.51	0.53	0.71
	-2.30	0.240							-0.233					3	-23.3	0.10	0.49	0.51	0.68
**3. Current abundance**																			
29	-1.10	0.073					0.076							3	-75.5	0.00	0.13	0.43	0.43
	-1.26	0.059	<0.001				0.088							4	-74.1	0.00	0.13	0.48	0.48
	-0.45		0.001				0.136			0.509	-0.105	-2.282	-0.566	7	-69.4	0.20	0.11	0.48	0.48
	-1.02	0.082												2	-77.0	0.58	0.09	0.63	0.63
	-0.15		<0.001				0.131			0.423				4	-74.4	0.64	0.09	0.37	0.37
	-1.20	0.075	<0.001											3	-76.1	1.33	0.06	0.47	0.47
	-1.17		0.001				0.152	0.012		0.487				5	-73.3	1.39	0.06	0.41	0.41
	-0.99	0.050	<0.001	-0.004			0.099							5	-73.4	1.50	0.06	0.51	0.51
	-1.00	0.043	<0.001				0.101			0.274				5	-73.4	1.52	0.06	0.51	0.51
	-0.84	0.058					0.087			0.247				4	-74.9	1.60	0.06	0.51	0.51
	-1.29	0.086							0.028					3	-76.3	1.64	0.05	0.45	0.45
	-1.29	0.076					0.068		0.021					4	-75.1	1.91	0.05	0.45	0.45
	-1.90	0.060	<0.001				0.100	0.008						5	-73.6	1.98	0.05	0.50	0.50

Online Resource 14 Continued

N	Int.	dia-meter	length	ground contact	bark	veget. cover	soft-ness	canopy	basal area	veget. type	stand 2	stand 3	stand 4	k	Log-Lik	ΔAICc	w_i	R^2_{LR}	R^2_N
**4. Long-term abundance**																			
40	-0.94	0.038				-0.009	0.125	0.029						5	-186.4	0.00	0.62	0.92	0.92
	-1.11	0.056					0.113	0.025		-0.391				5	-186.9	0.97	0.38	0.92	0.92
