# Supplementary material for: Microclimatic conditions mediate the effect of deadwood and forest characteristics on a threatened beetle species, Tragosoma depsarium
Source: Oecologia. 2022 Jul 11;199(3):737–52. doi: 10.1007/s00442-022-05212-w (PMC9309119; doi:10.1007/s00442-022-05212-w)
Supplement: Supplementary file 15 — Supplementary file15 (PDF 323 KB) [file 442_2022_5212_MOESM15_ESM.pdf]

Online Resource 15

Journal: Oecologia

Title: Microclimatic conditions mediate the effect of deadwood and forest characteristics on a threatened beetle species, *Tragosoma depsarium*

Authors: Ly Lindman, Erik Öckinger, Thomas Ranius

Corresponding author: L. Lindman, e-mail: Ly.Lindman@slu.se

Online Resource 15 Plausible candidate models (ΔAICc < 2) explaining current (1) and long-term (2) occurrence; and current (3) and long-term (4) abundance of *Tragosoma depsarium* in relation to microclimatic variables (t ºC – average temperature, t ºC fluct. – daily temperature fluctuations, max t ºC – maximum temperature, min t ºC – minimum temperature, RH % – relative humidity, aut. – autumn, sum. – summer). Sample size (N), intercept (Int.), number of parameters (k), model weight (w_i), a coefficient of determination based on the likelihood-ratio test (R^2_{LR}) and Nagelkerke's pseudo-R-squared (R^2_N) are presented

N	Int.	t ºC aut.	t ºC winter	t ºC spring	t ºC sum.	t ºC fluct. aut.	t ºC fluct. winter	t ºC fluct. spring	t ºC fluct. sum.	min t ºC winter	max t ºC sum.	RH % aut.	RH % winter	RH % spring	RH % sum.	k	Log-Lik	ΔAICc	w_i	R^2_{LR}	R^2_N
**1. Current occurrence**																					
55	7.10	-1.903					-2.770				0.365					4	-26.6	0.00	0.18	0.32	0.43
	6.50				0.580		-2.038									3	-27.9	0.27	0.16	0.29	0.39
	5.15				0.606		-2.022								-0.020	4	-27.1	0.84	0.12	0.31	0.42
	7.55	-2.184					-2.995				0.358		0.017			5	-25.9	0.98	0.11	0.34	0.46
	4.19				0.506		-2.322						0.024		-0.034	5	-26.0	1.19	0.10	0.34	0.46
	3.86						-2.651						0.031		-0.035	4	-27.4	1.48	0.09	0.30	0.41
	1.38						-2.358				0.227					3	-28.6	1.56	0.09	0.27	0.36
	0.60			0.434			-2.330									3	-28.7	1.78	0.08	0.27	0.36
	7.49	-1.772					-2.729				0.352				-0.010	5	-26.4	1.93	0.07	0.33	0.44
**2. Long-term occurrence**																					
55	1.59		-3.802	0.862												3	-22.9	0.00	0.18	0.40	0.54
	2.78		-4.286	1.090					-0.201							4	-22.0	0.50	0.14	0.42	0.57
	2.13		-3.686	1.079									-0.021			4	-22.1	0.78	0.12	0.42	0.57
	2.01			0.886			-2.527			-3.111						4	-22.2	0.94	0.11	0.42	0.56
	3.41		-4.214	1.343					-0.209				-0.023			5	-21.1	1.27	0.10	0.44	0.59
	7.29		-2.847		0.786											3	-23.6	1.44	0.09	0.39	0.52
	2.58		-3.901	0.973		-0.472										4	-22.5	1.49	0.09	0.41	0.56
	0.64		-4.082	1.176									-0.035		0.032	5	-21.3	1.50	0.09	0.44	0.59
	2.47			1.121			-2.508			-2.861			-0.022			5	-21.4	1.74	0.08	0.43	0.59

Online Resource 15 Continued

N	Int.	t ºC aut.	t ºC winter	t ºC spring	t ºC sum.	t ºC fluct. aut.	t ºC fluct. winter	t ºC fluct. spring	t ºC fluct. sum.	min t ºC winter	max t ºC sum.	RH % aut.	RH % winter	RH % spring	RH % sum.	k	Log-Lik	ΔAICc	w_i	R^2_{LR}	R^2_N
**3. Current abundance**																					
23	3.25				-0.199								0.011			3	-63.2	0.00	0.05	0.23	0.23
	1.89										-0.089			0.011		3	-63.2	0.03	0.05	0.23	0.23
	2.00										-0.080				0.009	3	-63.3	0.14	0.05	0.22	0.23
	2.81				-0.173									0.012		3	-63.3	0.19	0.05	0.22	0.22
	2.86				-0.157										0.010	3	-63.3	0.21	0.05	0.22	0.22
	0.36														0.009	2	-64.7	0.21	0.05	0.13	0.13
	2.11										-0.096		0.010			3	-63.3	0.23	0.05	0.22	0.22
	1.02					-0.258									0.010	3	-63.6	0.64	0.04	0.21	0.21
	2.80										-0.080					2	-64.9	0.68	0.04	0.11	0.11
	0.17													0.010		2	-64.9	0.68	0.04	0.11	0.11
	0.80						-0.368								0.009	3	-63.6	0.73	0.04	0.20	0.21
	1.14															1	-66.2	0.92	0.03	0.00	0.00
	0.81					-0.273								0.012		3	-63.7	0.96	0.03	0.20	0.20
	3.53				-0.147											2	-65.1	1.01	0.03	0.10	0.10
	0.61						-0.387							0.010		3	-63.8	1.10	0.03	0.19	0.19
	1.60						-0.385									2	-65.2	1.27	0.03	0.09	0.09
	0.31												0.008			2	-65.2	1.29	0.03	0.08	0.09
	0.97					-0.289							0.010			3	-63.9	1.33	0.03	0.18	0.18
	2.85	-0.504											0.011			3	-64.0	1.53	0.02	0.18	0.18
	2.32	-0.412												0.011		3	-64.0	1.59	0.02	0.17	0.18
	3.21				-0.173		-0.312						0.011			4	-62.6	1.60	0.02	0.27	0.27
	0.75						-0.395						0.009			3	-64.0	1.63	0.02	0.17	0.17
	2.18	-0.329													0.009	3	-64.1	1.71	0.02	0.17	0.17

Online Resource 15 Continued

N	Int.	t ºC aut.	t ºC winter	t ºC spring	t ºC sum.	t ºC fluct. aut.	t ºC fluct. winter	t ºC fluct. spring	t ºC fluct. sum.	min t ºC winter	max t ºC sum.	RH % aut.	RH % winter	RH % spring	RH % sum.	k	Log-Lik	ΔAICc	w_i	R^2_{LR}	R^2_N
	0.76								-0.046						0.009	3	-64.1	1.75	0.02	0.17	0.17
	2.78				-0.147		-0.316							0.012		4	-62.6	1.76	0.02	0.27	0.27
	2.83				-0.132		-0.306								0.010	4	-62.7	1.80	0.02	0.27	0.27
	0.54							-0.063						0.012		3	-64.1	1.81	0.02	0.17	0.17
	0.67		-0.350												0.010	3	-64.1	1.83	0.02	0.17	0.17
	1.75					-0.205										2	-65.5	1.88	0.02	0.17	0.17
	0.57								-0.051					0.010		3	-64.2	1.94	0.02	0.27	0.27
	0.74							-0.047							0.009	3	-64.2	1.96	0.02	0.27	0.27
	3.32	-0.512					-0.403						0.012			4	-62.8	2.00	0.02	0.26	0.26
**4. Long-term abundance**																					
33	3.69		-0.725	0.102								-0.025	0.014			5	-138.4	0.00	1.00	0.95	0.95
